# Utility of Biomarkers for Sepsis-Associated Acute Kidney Injury Staging

**DOI:** 10.1001/jamanetworkopen.2022.12709

**Published:** 2022-05-18

**Authors:** Luca Molinari, Gaspar Del Rio-Pertuz, Ali Smith, Douglas P. Landsittel, Kai Singbartl, Paul M. Palevsky, Lakhmir S. Chawla, David T. Huang, Donald M. Yealy, Derek C. Angus, John A. Kellum

**Affiliations:** 1 Center for Critical Care Nephrology, Department of Critical Care Medicine, School of Medicine, University of Pittsburgh, Pittsburgh, Pennsylvania; 2 Department of Translational Medicine, Università degli Studi del Piemonte Orientale, Novara, Italy; 3 Department of Internal Medicine, Texas Tech University Health Sciences Center, Lubbock; 4 Clinical Research, Investigation, and Systems Modeling of Acute Illness Center, Department of Critical Care Medicine, School of Medicine, University of Pittsburgh, Pittsburgh, Pennsylvania; 5 Department of Biomedical Informatics, School of Medicine, University of Pittsburgh, Pittsburgh, Pennsylvania; 6 Department of Critical Care Medicine, Mayo Clinic, Phoenix, Arizona; 7 Kidney Medicine (Renal) Section, Medical Service, Veterans Affairs Pittsburgh Healthcare System, Pittsburgh, Pennsylvania; 8 Renal-Electrolyte Division, Department of Medicine, University of Pittsburgh, Pittsburgh, Pennsylvania; 9 Department of Medicine, Veterans Affairs Medical Center, San Diego, California; 10 Department of Emergency Medicine, School of Medicine, University of Pittsburgh, Pittsburgh, Pennsylvania

## Abstract

**Importance:**

The 23rd Acute Disease Quality Initiative (ADQI-23) consensus conference proposed a framework to integrate biomarkers into the staging of acute kidney injury (AKI). It is unknown whether tissue inhibitor of metalloproteinases 2 (TIMP-2) and insulinlike growth factor binding protein 7 (IGFBP7) could be used for staging.

**Objective:**

To test whether higher levels of urinary [TIMP-2] × [IGFBP7] are associated with lower survival among patients with the same functional stage of AKI.

**Design, Setting, and Participants:**

This cohort study was performed using data from the Protocolized Care for Early Septic Shock (ProCESS) trial, which enrolled critically ill patients with septic shock who presented at academic and community emergency departments and intensive care units in the US from March 2008 to May 2013. Patients with end-stage kidney disease, a reference serum creatinine level of 4 mg/dL or greater (to convert to μmol/L, multiply by 76.25), or missing data on serum creatinine levels or urinary levels of [TIMP-2] × [IGFBP7] were excluded. Data were analyzed from October 2020 to October 2021.

**Exposures:**

The presence of AKI, assessed using Kidney Disease: Improving Global Outcomes criteria within 24 hours after enrollment and the highest AKI stage as well as urinary [TIMP-2] × [IGFBP7] level at 6 hours after enrollment. A previously reported high-specificity cutoff level for [TIMP-2] × [IGFBP7] of 2.0 (ng/mL)^2^/1000 was used to categorize patients (including those without functional criteria of AKI) according to the new staging system proposed by the ADQI-23 as biomarker negative (urinary [TIMP-2] × [IGFBP7] level ≤2.0 [ng/mL]^2^/1000) or biomarker positive ([TIMP-2] × [IGFBP7] >2.0 [ng/mL]^2^/1000).

**Main Outcomes and Measures:**

Survival (assessed using Kaplan-Meier plots and the log-rank test) and mortality (assessed using relative risk [RR] 30 days after enrollment).

**Results:**

The analysis included 999 patients with a median age of 61 years (IQR, 50-73 years); 554 (55.5%) were male. Biomarker-positive patients had lower survival and higher mortality at 30 days in the groups with AKI stage 1 (RR, 2.20; 95% CI, 1.02-4.72), stage 2 (RR, 1.53; 95% CI, 1.04-2.27), and stage 3 (RR, 1.61; 95% CI, 1.00-2.60). The associations were specific to patients with AKI. No difference in 30-day survival was found between biomarker-positive and biomarker-negative patients in the absence of functional criteria for AKI (RR, 1.16; 95% CI, 0.45-3.01).

**Conclusions and Relevance:**

The findings suggest that assessment of the cell-cycle arrest biomarkers TIMP-2 and IGFBP7 may augment AKI staging for patients with functional criteria for AKI.

## Key Points

**Question:**

Can measuring the cell-cycle arrest biomarkers tissue inhibitor of metalloproteinases 2 (TIMP-2) and insulinlike growth factor binding protein 7 (IGFBP7) enhance acute kidney injury (AKI) staging using a recently proposed framework?

**Findings:**

In this cohort study of 999 participants from a randomized clinical trial of critically ill patients with septic shock, in patients who developed AKI within 24 hours after enrollment, a urinary [TIMP-2] × [IGFBP7] level greater than 2.0 (ng/mL)^2^/1000 was associated with greater mortality risk at 30 days when AKI stages were defined by kidney function.

**Meaning:**

The findings suggest that integrating cell-cycle arrest biomarkers in a new staging framework for AKI may improve discrimination for survival.

## Teaser

This cohort study examines associations between urinary levels of 2 cell-cycle arrest biomarkers and 30-day survival among patients with septic shock who developed acute kidney injury staged based on kidney function.

## Introduction

Acute kidney injury (AKI) is defined by an abrupt decrease of the glomerular filtration rate and has multiple causes. Surrogates for the glomerular filtration rate, specifically serum creatinine level and urine output, are used to stage AKI severity according to Kidney Disease: Improving Global Outcomes (KDIGO) criteria.^1^ Although there is good correlation between AKI stage and histologic findings on kidney biopsy,^2^ there can be kidney damage without change in function^3^ and injury not detected by AKI criteria. Furthermore, as in chronic kidney disease, the presence or absence of damage markers might add to severity grading in AKI when functional changes are also present.^4^ Several biomarkers of kidney stress (that can revert or progress to damage or to altered kidney function) and damage (that may or may not be associated with reduced kidney function) have been evaluated in the past 15 years; these markers could be constitutive proteins released by the damaged kidney, molecules upregulated in response to injury, or nonkidney tissue products that are filtered, reabsorbed, or secreted by the kidney.^5,6,7,8,9,10^ However, their evaluation and commercial development have been focused on predicting functional change, not on assessing damage or augmenting AKI staging. The only US Food and Drug Administration–approved test for AKI, NephroCheck Test (Astute Medical, Inc), measures 2 urinary biomarkers, tissue inhibitor of metalloproteinases 2 (TIMP-2) and insulinlike growth factor binding protein 7 (IGFBP7), combining the product of the concentration of the 2 biomarkers in the score [TIMP-2] × [IGFBP7].^11^ These molecules are released from kidney tubular cells even with sublethal noxious stimuli, such as a nutrient deprivation in vitro^12^ or remote ischemic preconditioning in vivo.^13^ Whether such stress markers identify damage at higher thresholds is uncertain.

In 2020, the 23rd Acute Disease Quality Initiative (ADQI-23) work group proposed an expanded classification for AKI, adding biomarkers for each stage (Table 1).^10,14^ Existing evidence suggests that among patients who have already developed AKI, an elevated [TIMP-2] × [IGFBP7] level may identify patients at higher risk of severe AKI, death, or need for kidney replacement therapy compared with patients without elevated biomarkers.^15,16,17^ Moreover, compared with biomarker-negative patients without standard functional criteria for AKI, higher levels of [TIMP-2] × [IGFBP7] are associated with a reduction in kidney functional reserve.^18^ The ADQI-23 work group specified the criteria for qualifying biomarkers to aid in the classification of AKI (rather than to detect it).^10^ In brief, a biomarker candidate must differentiate patients with a different prognosis at a given functional stage. In this cohort study of critically ill patients with septic shock from the Protocolized Care for Early Septic Shock (ProCESS) trial,^19^ we explored whether higher levels of [TIMP-2] × [IGFBP7], measured nearly simultaneously with assessment of AKI, could identify patients with reduced survival among those at the same functional AKI stage.

**Table 1.** Proposed New Definition and Staging of Acute Kidney Injury by the ADQI-23 Consensus Conference^a^

KDIGO stage	Functional criteria	Biomarkers	New stage
No AKI	No increased sCr level (≥0.3 mg/dL) in ≤48 h and No increased sCr level (≥1.5 mg/dL from baseline) in 7 d and UO >0.5 mL/kg/h in 6-h period	Negative	No AKI
		Positive	1S
1	Increased sCr level (≥0.3 mg/dL) in ≤48 h or Increased sCr level (1.5-1.9 times baseline) in <7 d or UO <0.5 mL/kg/h for 6-12 h	Negative	1A
		Positive	1B
2	Increased sCr level (2.0-2.9 times baseline) or UO <0.5 mL/kg/h for ≥12 h	Negative	2A
		Positive	2B
3	Increased sCr level (≥3.0 times baseline) or sCr level ≥4.0 mg/dL with acute increase of ≥0.3 mg/dL or UO <0.3 mL/kg/h for ≥24 h or Anuria for ≥12 h or Initiation of kidney replacement therapy	Negative	3A
		Positive	3B

Abbreviations: ADQI-23, 23rd Acute Disease Quality Initiative; AKI, acute kidney injury; KDIGO, Kidney Disease: Improving Global Outcomes; sCr, serum creatinine; UO, urine output.

SI conversion factor: To convert serum creatinine to micromoles per liter, multiply by 76.25.

^a^ Adapted from the ADQI-23^14^ and published by Ostermann et al.^10^

## Methods

### Study Design and Patients

This cohort study used existing data from the ProCESS trial. The ProCESS trial design, methods, and main results have been described elsewhere.^19,20^ The ProCESS trial was a multicenter, randomized clinical trial of 3 different resuscitation strategies in patients with septic shock that enrolled 1341 patients at academic and community emergency departments and intensive care units in the US from March 2008 to May 2013. We excluded patients with end-stage kidney disease (receiving long-term dialysis not associated with the current sepsis episode), a reference serum creatinine level of 4 mg/dL or greater (to convert to μmol/L, multiply by 76.25), missing serum creatinine level at admission, or missing measurement of urinary [TIMP-2] × [IGFBP7] (eAppendix 1 and eFigure 1 in Supplement 1). This retrospective study was performed in accordance with the 1964 Declaration of Helsinki.^21^ The Office of Human Research Protection at the University of Pittsburgh approved this ancillary analysis, which was conducted using deidentified data. All patients or their legally authorized representatives provided written informed consent. This study followed the Strengthening the Reporting of Observational Studies in Epidemiology (STROBE) reporting guideline. Data were analyzed from October 2020 to October 2021.

We collected patient demographics, health history, and serum creatinine level (12 months before and at enrollment and then daily until discharge from the hospital or truncated at 28 days) and urine output (hourly for the first 72 hours or until discharge from the intensive care unit) values as previously reported.^19^ We classified AKI according to KDIGO criteria using both serum creatinine level and urine output (eAppendix 2 in Supplement 1).^1^ We obtained urine samples for biomarker testing at 6 hours after enrollment and the start of resuscitation. We centrifuged the samples immediately after collection and froze and stored the supernatant at less than –70 °C. We thawed the supernatant immediately before testing for the [TIMP-2] × [IGFBP7] level with the NephroCheck Test. Tests were performed according to the manufacturer’s specifications.

### Exposures and End Points

We evaluated the presence of AKI based on KDIGO criteria between 0 and 24 hours after enrollment and the highest AKI stage and the urinary [TIMP-2] × [IGFBP7] level at 6 hours after enrollment. To augment AKI diagnosis and staging, we applied a previously reported high-specificity cutoff for [TIMP-2] × [IGFBP7] of 2.0 (ng/mL)^2^/1000^15,22^ to categorize patients according to the new AKI staging system proposed by ADQI-23 (Table 1).^10^

Our primary end point was survival 30 days after enrollment. Our secondary end points were (1) all-cause mortality 30 days after enrollment; (2) full recovery from AKI (for patients who developed AKI at any stage in the first 24 hours), defined as the absence of any AKI on the last day available (truncated at 28 days after enrollment); (3) AKI stage 3 by day 7 (for patients who did not develop AKI stage 3 in the first 24 hours), defined as reaching AKI stage 3 in any day between day 1 (excluded) and day 7 (included) after enrollment; and (4) hospital length of stay (LOS), truncated at 60 days after enrollment among patients who were alive at 60 days.

### Statistical Analysis

We compared survival 30 days after enrollment among patients at the new AKI stages obtained with the [TIMP-2] × [IGFBP7] level using Kaplan-Meier plots and the log-rank test. We also used Cox proportional hazards regression models to obtain adjusted hazard ratios (HRs) for death within 30 days and corresponding adjusted survival curves. To adjust survival curves, we used covariates present before the occurrence of the exposures, such as age, sex, race and ethnicity, and Charlson Comorbidity Index (to assess the burden of previous comorbidities).

For the secondary end points, we expressed categorical variables as absolute numbers and percentages and compared them using Pearson χ^2^ or Fisher exact tests together with relative risk (RR) (95% CI) as an effect measure. We expressed continuous variables as medians and IQRs and compared them using the Mann-Whitney *U* test together with the independent-samples Hodges-Lehmann median difference (95% CI) as an effect measure. We did not apply corrections for multiple comparisons regarding the 3 secondary end points because they were considered as exploratory. We conducted all analyses using SPSS, version 26 (IBM Corp), and we set the per-comparison significance at 2-sided *P* < .05.

We performed a sensitivity analysis evaluating associations between the end points and cutoffs of 0.3 and 1.0 (ng/mL)^2^/1000 for the [TIMP-2] × [IGFBP7] level. The 0.3 (ng/mL)^2^/1000 cutoff is considered a high-sensitivity cutoff based on the results of both its derivation^22^ and validation studies,^23^ whereas the 1.0 (ng/mL)^2^/1000 cutoff has been evaluated for sepsis and has shown good sensitivity and specificity compared with the 0.3 and 2.0 (ng/mL)^2^/1000 cutoffs^24^ (eAppendix 3 in Supplement 1).

## Results

The analysis cohort included 999 patients for whom the [TIMP-2] × [IGFBP7] level at 6 hours was available (eFigure 1 in Supplement 1); the median age was 61 years (IQR, 50-73 years), and 554 (55.5%) were male (Table 2 gives general characteristics). Characteristics of the overall cohort of 1243 patients, including the 244 who were excluded, were similar (eTable 1 in Supplement 1). A total of 626 patients (62.7%) developed AKI within 24 hours after enrollment. With use of standard KDIGO staging based on both serum creatinine level and urine output, 152 patients (15.2%) had a maximum AKI stage of 1, 294 (29.4%) had stage 2; and 180 (18.0%) had stage 3 (eAppendix 2 in Supplement 1). Within a 7-day time window, 686 patients (68.7%) developed AKI; 540 (78.7%) of these patients reached the maximum AKI KDIGO stage within the first 24 hours. A total of 193 patients (19.3%) died within 30 days.

**Table 2.** General Characteristics of the Analysis Cohort

Characteristic	Participants (N = 999)^a^
Age, median (IQR), y	61 (50-73)
Sex	
Female	445 (44.5)
Male	554 (55.5)
Race and ethnicity^b^	
African American/Black	234 (23.4)
White	695 (69.6)
Other^c^	70 (7.0)
Cardiovascular disease	650 (65.1)
Arterial hypertension	584 (58.5)
Congestive heart failure	111 (11.1)
Previous myocardial infarction	108 (10.8)
Cerebral vascular disease	105 (10.5)
Peripheral vascular disease	75 (7.5)
Diabetes	333 (33.3)
Chronic respiratory disease	234 (23.4)
Kidney disease history	101 (10.1)
Active cancer	179 (17.9)
Dementia	78 (7.8)
Cirrhosis	59 (5.9)
Peptic ulcer disease	54 (5.4)
HIV infection	25 (2.5)
Charlson Comorbidity Index score, median (IQR)	2 (1-4)
SOFA score at enrollment, median (IQR)	7 (4-9)
APACHE II score at enrollment, median (IQR)	19 (15-24)
AKI status 0-24 h after enrollment	
AKI	629 (62.7)
No AKI	373 (37.3)
KDIGO stage	
1	152 (15.2)
2	294 (29.4)
3	180 (18.0)
Mortality at 30 d	193 (19.3)
Hospital LOS, median (IQR), d	8 (5-14)
[TIMP-2] × [IGFBP7] level at 6 h, (ng/mL)^2^/1000	
Median (IQR)	0.35 (0.12-1.41)
>2.0	196 (19.6)
>1.0	291 (29.1)
>0.3	538 (53.9)

Abbreviations: AKI, acute kidney injury; APACHE, Acute Physiology and Chronic Health Evaluation; IGFBP7, insulinlike growth factor binding protein 7; KDIGO, Kidney Disease: Improving Global Outcomes; LOS, length of stay; SOFA, Sequential Organ Failure Assessment; TIMP-2, tissue inhibitor of metalloproteinases 2.

^a^ Data are presented as number (percentage) of participants unless otherwise indicated.

^b^ Race and ethnicity was determined by patient self-report or by the patient’s legally authorized representative.

^c^ Other includes Asian, American Indian or Native Alaskan, Native Hawaiian or other Pacific Islander, unknown, or other.

The median [TIMP-2] × [IGFBP7] level was 0.35 (ng/mL)^2^/1000 (IQR, 0.12-1.41 [ng/mL]^2^/1000), and a total of 196 patients (19.6%) had a [TIMP-2] × [IGFBP7] level greater than 2.0 (ng/mL)^2^/1000. The median time between reaching the maximum AKI stage (in the 7-day time window) and biomarker measurement was −6 hours (IQR, −6 to 18 hours). Among those alive at 60 days, the median hospital LOS was 8 days (IQR, 5-14 days). The LOS was greater among patients with a [TIMP-2] × [IGFBP7] level greater than 2.0 (ng/mL)^2^/1000 compared with 2.0 (ng/mL)^2^/1000 or less (11 days [IQR, 7-20 days] vs 8 days [IQR, 5-13 days]; *P* < .001), and among patients with standard KDIGO criteria for AKI (pooling stages 1-3), the presence of a [TIMP-2] × [IGFBP7] level greater than 2.0 (ng/mL)^2^/1000 was associated with a longer hospital LOS than among patients with a [TIMP-2] × [IGFBP7] level of 2.0 (ng/mL)^2^/1000 or less (11 days [IQR, 7-20 days] vs 9 days [IQR, 6-14 days]; *P* = .008).

### AKI Staging Using [TIMP-2] × [IGFBP7] and Survival

**Figure 1.**
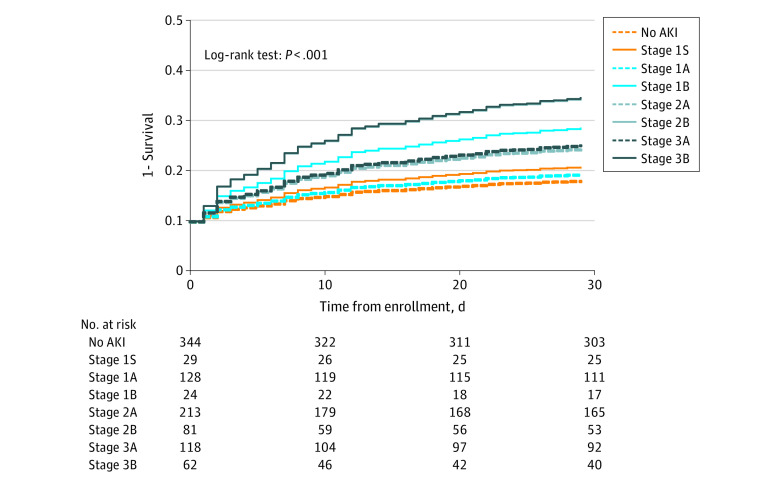
Covariate-Adjusted Survival by New Acute Kidney Injury (AKI) Stage Adjusted survival curves for the new AKI stages based on whether the product of tissue inhibitor of metalloproteinases 2 × insulinlike growth factor binding protein 7 level was 2.0 (ng/mL)^2^/1000 or less or greater than 2.0 (ng/mL)^2^/1000. Details about the definition of stages are in Table 1. Covariates used in the Cox proportional hazards regression models were age, sex, race and ethnicity, and Charlson Comorbidity Index. Dashed lines indicate patients who were biomarker negative, and solid lines indicate those who were biomarker positive.

**Figure 2.**
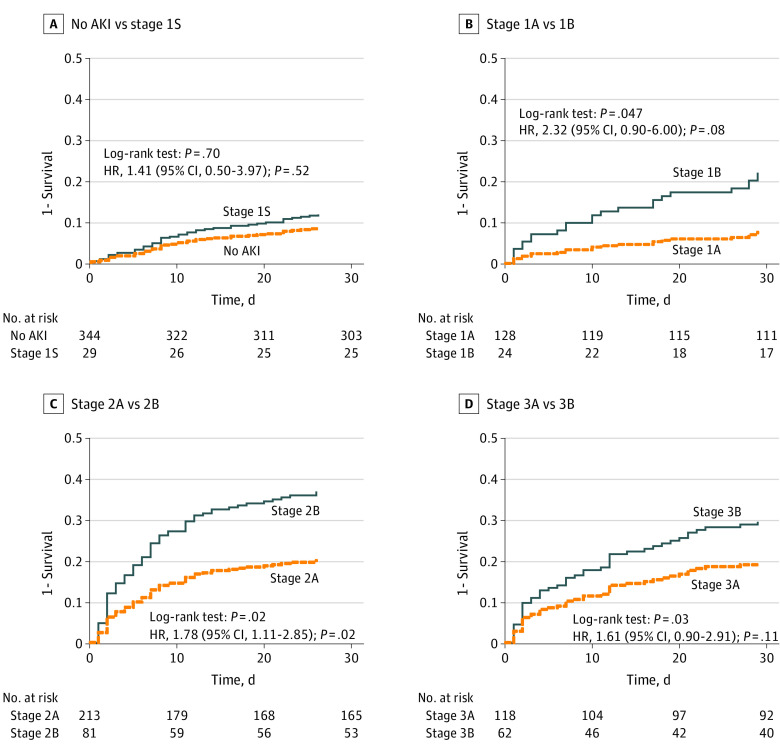
Covariate-Adjusted Survival Among Patients at Each Functional Acute Kidney Injury (AKI) Stage Each plot shows patients with the same Kidney Disease: Improving Global Outcomes functional AKI stage discriminating covariate-adjusted survival according to the presence of a tissue inhibitor of metalloproteinases 2 × insulinlike growth factor binding protein 7 level of 2.0 (ng/mL)^2^/1000 or less or greater than 2.0 (ng/mL)^2^/1000. For details about the definition of stages, refer to Table 1. Covariates used in the Cox proportional hazards regression models were age, sex, race and ethnicity, and Charlson Comorbidity Index. Dashed lines indicate patients who were biomarker negative, and solid lines indicate those who were biomarker positive.

We applied the [TIMP-2] × [IGFBP7] cutoff of 2.0 (ng/mL)^2^/1000 to each functional KDIGO AKI stage. Among patients with no AKI, 29 of 373 (7.8%) had a [TIMP-2] × [IGFBP7] level greater than 2.0 (ng/mL)^2^/1000 (new stage 1S, as shown in Table 1). Among patients with AKI stage 1, 24 of 152 (15.8%) had a [TIMP-2] × [IGFBP7] level greater than 2.0 (ng/mL)^2^/1000 (new stage 1B). For AKI stage 2, 81 of 294 patients (27.6%) had a [TIMP-2] × [IGFBP7] level greater than 2.0 (ng/mL)^2^/1000 (new stage 2B), and for AKI stage 3, 62 of 180 (34.4%) had a [TIMP-2] × [IGFBP7] level greater than 2.0 (ng/mL)^2^/1000 (new stage 3B). The general characteristics of each stage are reported in eTable 2 in Supplement 1. Survival was different across all 8 stages obtained with the [TIMP-2] × [IGFBP7] level (Figure 1 shows the corresponding covariate-adjusted survival curves). With use of no AKI as the reference, the HRs were 1.36 (95% CI, 0.49-3.81; *P* = .56) for stage 1S, 1.16 (95% CI, 0.66-2.06; *P* = .60) for stage 1A, 2.45 (95% CI, 1.09-5.52; *P* = .03) for stage 1B, 1.85 (95% CI, 1.22-2.82; *P* = .004) for stage 2A, 3.38 (95% CI, 2.08-5.49; *P* < .001) for stage 2B, 1.95 (95% CI, 1.19-3.20; *P* = .008) for stage 3A, and 3.41 (95% CI, 2.02-5.75; *P* < .001) for stage 3B. Pairwise comparisons for survival within the same functional stage using the log-rank test were statistically different for stage 1A vs 1B (Figure 2B), stage 2A vs 2B (Figure 2C), and stage 3A vs 3B (Figure 2D). Survival among patients with no AKI was not different from that among patients with stage 1S (Figure 2A). However, covariate-adjusted survival was significantly different for stage 2B compared with stage 2A (HR, 1.78; 95% CI, 1.11-2.85; *P* = .02) (Figure 2C).

### AKI Staging Using [TIMP-2] × [IGFBP7] and Secondary End Points

Table 3 summarizes the secondary end points according to each KDIGO stage and compares them within each stage between [TIMP-2] × [IGFBP7] levels of 2.0 (ng/mL)^2^/1000 or less vs greater than 2.0 (ng/mL)^2^/1000. All-cause mortality at 30 days was greater for stage 1B vs 1A, with corresponding mortality rates of 29.2% (95% CI, 14.1%-48.9%) vs 13.3% (95% CI, 8.2%-20.0%) (*P* = .050 for χ^2^ test) and an RR of 2.20 (95% CI, 1.02-4.72). For stage 2B vs 2A, the corresponding mortality rates were 34.6% (95% CI, 24.9%-45.3%) vs 22.5% (95% CI, 17.3%-28.5%) (*P* = .04) and the RR was 1.53 (95% CI, 1.04-2.27). For stage 3B vs 3A, the corresponding mortality rates were 35.5% (95% CI, 24.5%-47.8%) vs 22.0% (15.3%-30.1%) (*P* = .052) and the RR was 1.61 (95% CI, 1.00-2.60). Mortality was similar for stage 1S compared with no AKI, with a corresponding mortality rate of 13.8% (95% CI, 4.8%-29.5%) vs 11.9% (95% CI, 8.8%-15.7%) (*P* = .77) and an RR of 1.16 (95% CI, 0.45-3.01), but hospital LOS was significantly longer for patients with stage 1S, with a median of 11 days (IQR, 8-18 days) vs 7 days (IQR, 5-11 days) (*P* = .003). Similarly, for stage 2, patients with a [TIMP-2] × [IGFBP7] level greater than 2.0 (ng/mL)^2^/1000 had a longer hospital LOS, with a median of 13 days (IQR, 8-21 days) for patients with stage 2B and 8 days (IQR, 6-14 days) for patients with stage 2A (*P* = .001). With regard to full recovery from AKI, only patients with stage 2B showed a lower rate of recovery (49.4%; 95% CI, 38.7%-60.1%) compared with patients with stage 2A (70.9%; 95% CI, 64.5%-76.7%) (*P* = .001).

**Table 3.** Secondary End Points Compared Between Participants Within the Same Functional KDIGO AKI Stage Who Were Negative vs Positive for Biomarkers

End point	Estimate^a^			Relative risk or median difference (95% CI)^b^	*P* value^c^
	KDIGO stage	[TIMP-2] × [IGFBP7] level ≤2.0 (ng/mL)^2^/1000	[TIMP-2] × [IGFBP7] level >2.0 (ng/mL)^2^/1000		
	**No AKI (n = 373)**	**No AKI (n = 344)**	**Stage 1S (n = 29)**		
Mortality at 30 d	12.1 (9.1 to 15.7)	11.9 (8.8 to 15.7)	13.8 (4.8 to 29.5)	1.16 (0.45 to 3.01)	.77
Full recovery	NA	NA	NA	NA	NA
AKI stage 3 by day 7	4.0 (2.4 to 6.4)	4.1 (2.4 to 6.6)	3.4 (0.4 to 15.0)	0.85 (0.12 to 6.22)	.87
Hospital LOS, d	7 (5 to 12)	7 (5 to 11)	11 (8 to 18)	3 (1 to 6)	.003
	**Stage 1 (n = 152)**	**Stage 1A (n = 128)**	**Stage 1B (n = 24)**		
Mortality at 30 d	15.8 (10.7 to 22.2)	13.3 (8.2 to 20.0)	29.2 (14.1 to 48.9)	2.20 (1.02 to 4.72)	.050
Full recovery	80.3 (73.4 to 86.0)	81.3 (73.8 to 87.3)	75 (55.5 to 88.8)	0.92 (0.72 to 1.18)	.48
AKI stage 3 by day 7	5.3 (2.5 to 9.7)	3.9 (1.5 to 8.3)	12.5 (3.6 to 29.7)	3.20 (0.82 to 12.51)	.08
Hospital LOS, d	9 (6 to 13)	9 (6 to 12)	10 (7 to 16)	1 (–2 to 4)	.42
	**Stage 2 (n = 294)**	**Stage 2A (n = 213)**	**Stage 2B (n = 81)**		
Mortality at 30 d	25.9 (21.1 to 31.1)	22.5 (17.3 to 28.5)	34.6 (24.9 to 45.3)	1.53 (1.04 to 2.27)	.04
Full recovery	65 (59.4 to 70.3)	70.9 (64.5 to 76.7)	49.4 (38.7 to 60.1)	0.70 (0.55 to 0.88)	.001
AKI stage 3 by day 7	19.4 (15.2 to 24.2)	16.9 (12.3 to 22.4)	25.9 (17.3 to 36.2)	1.53 (0.96 to 2.46)	.08
Hospital LOS, d	10 (6 to 15)	8 (6 to 14)	13 (8 to 21)	4 (1 to 6)	.001
	**Stage 3 (n = 180)**	**Stage 3A (n = 118)**	**Stage 3B (n = 62)**		
Mortality at 30 d	26.7 (20.6 to 33.5)	22.0 (15.3 to 30.1)	35.5 (24.5 to 47.8)	1.61 (1.00 to 2.60)	.052
Full recovery	47.8 (40.6 to 55.1)	51.7 (42.7 to 60.6)	40.3 (28.8 to 52.7)	0.78 (0.55 to 1.11)	.15
AKI stage 3 by day 7	NA	NA	NA	NA	NA
Hospital LOS, d	11 (7 to 20)	11 (7 to 19)	9 (7 to 22)	0 (–3 to 3)	.90

Abbreviations: AKI, acute kidney injury; IGFBP7, insulinlike growth factor binding protein 7; KDIGO, Kidney Disease: Improving Global Outcomes; LOS, length of stay; NA, not applicable; TIMP-2, tissue inhibitor of metalloproteinases 2.

^a^ Categorical variables are presented as percentage of patients (95% CI) and continuous variables as median (IQR).

^b^ Based on independent-samples Hodges-Lehmann median differences. Relative risks are reported for categorial variables and median differences for continuous variables.

^c^ *P* values are from the χ^2^ test or Mann-Whitney *U* test as appropriate.

### Sensitivity Analysis

eAppendix 3 in Supplement 1 gives the results of the sensitivity analysis using alternative [TIMP-2] × [IGFBP7] cutoff levels of 1.0 and 0.3 (ng/mL)^2^/1000. In brief, the 1.0 (ng/mL)^2^/1000 cutoff differentiated 30-day survival between no AKI and stage 1S and between stages 2A and 2B (eFigures 2 and 3 and eTable 3 in Supplement 1), whereas the 0.3 (ng/mL)^2^/1000 cutoff differentiated survival only between stages 2A and 2B (eFigures 4 and 5 and eTable 4 in Supplement 1).

## Discussion

Many investigators have suggested implementing biomarkers in the evaluation of AKI,^8,9,25^ and the ADQI-23 consensus conference^10^ provided a conceptual framework for expanding functional classification of AKI by adding biomarkers. The cell-cycle arrest biomarkers TIMP-2 and IGFBP7 are produced by kidney tubule epithelial cells in response to kidney stress.^5,9^ A [TIMP-2] × [IGFBP7] level greater than 2.0 (ng/mL)^2^/1000 has high specificity (95%) for predicting moderate to severe AKI^23^; thus, we used this cutoff for our primary analysis. Lower cutoffs of 0.3 and 1.0 (ng/mL)^2^/1000 have been reported in the literature^22,23,24^; they have higher sensitivity for detecting AKI, but for staging a disease or syndrome, high specificity is needed. For this reason, we chose 2.0 (ng/mL)^2^/1000 as the cutoff for our primary analysis.

The ADQI-23 consensus recommendations emphasized the need for biomarkers to add prognostic information to existing function-based AKI staging.^10^ However, it is important that this information be specific to AKI rather than an overall prognostic marker. In this study, a [TIMP-2] × [IGFBP7] level greater than 2.0 (ng/mL)^2^/1000 measured nearly simultaneously with the assessment of AKI was associated with lower survival among patients at all 3 AKI stages but not among patients without AKI (stage 1S), although patients in this group had a longer median hospital LOS if their [TIMP-2] × [IGFBP7] level was greater than 2.0 (ng/mL)^2^/1000. Similar to these findings, prior studies showed that the rate of death or dialysis by 9 months^15,17^ or by hospital discharge^16^ was increased in patients positive for a biomarker only when functional changes also occurred. As such, the prognostic signal would seem to be specific to AKI and would not be a general mortality indicator. The results are also consistent with the nature of TIMP-2 and IGFBP7 as stress rather than damage biomarkers. Stress in isolation may be benign, but when it coexists with dysfunction, it may signal actual damage.^17^ However, this interpretation is challenged by the work of Husain-Syed and colleagues,^18^ who demonstrated that an increased urinary [TIMP-2] × [IGFBP7] level after cardiac surgery was associated with loss of kidney functional reserve at 3 months even in the absence of AKI. Thus, crude measures, such as death or dialysis, may not help detect subclinical injury, but measuring kidney functional reserve may help reveal it. It is also possible that in certain conditions (eg, loss of muscle mass), creatinine levels may fail to accurately reflect kidney function. With regard to our secondary end points, we also found that for certain functional stages (no AKI and stage 2), a [TIMP-2] × [IGFBP7] level greater than 2.0 (ng/mL)^2^/1000 was associated with a longer hospital LOS and, only for patients with stage 2, a lower rate of full recovery from AKI. However, caution must be used when interpreting the results of the secondary end points because they were exploratory and should only be used to guide future studies using more kidney-related end points.

To our knowledge, this is the first study to specifically examine the conceptual framework recently put forth by the ADQI-23 work group. However, we are not the first to examine how biomarkers might add information to standard functional stages. Uhel and colleagues^26^ examined 16 plasma biomarkers reflecting pathways involved in sepsis pathogenesis and blood leukocyte transcriptomes in patients with sepsis. They found associations between these biomarker signatures and both AKI occurrence and persistence, as defined by ADQI-16,^27^ finding significant differences in both. We chose to focus on the only commercially available biomarker test for AKI in the US because it is also available worldwide and has a published high-specificity cutoff and because an ongoing trial is already using it specifically for patients with sepsis.^28^ Future work is needed to evaluate other potential biomarkers alone or in combination to augment AKI staging, such as kidney injury molecule 1 and neutrophil gelatinase–associated lipocalin. Prior evidence suggests that kidney injury molecule 1 may be able to detect kidney damage below the serum creatinine threshold^7,29,30^ and that patients positive for neutrophil gelatinase–associated lipocalin, even without the presence of serum creatinine criteria for AKI, were at greater risk of death and/or kidney replacement therapy.^31,32^ Our sensitivity analysis supported our contention that a low [TIMP-2] × [IGFBP7] cutoff (0.3 [ng/mL]^2^/1000) may not be useful for substaging AKI. However, the 1.0 (ng/mL)^2^/1000 cutoff may deserve further investigation because it discriminated outcomes by functional AKI stage nearly as well as the 2.0 (ng/mL)^2^/1000 cutoff but also discriminated in the absence of functional criteria (no AKI vs stage 1S), a condition in which the 2.0 (ng/mL)^2^/1000 cutoff failed to show a difference in survival.

### Limitations

This study has limitations. First, this was a retrospective analysis of data previously collected from a randomized clinical trial and data did not come from an effort specifically designed to test our hypothesis. Although this allowed us to access a large set of clinical data with timely sample collection and standardized definitions of AKI and clinical outcomes, the database did not have biomarker results for all patients. Excluding patients with missing biomarker values may have caused a selection bias by excluding patients with a potentially more severe condition (eg, anuria, early mortality). Second, even if sepsis accounts for almost half of the cases of AKI in critically ill patients,^33^ patients in this study had community-acquired septic shock; thus, our results may not be generalizable to other AKI causes. Third, the timing for the measurements of urinary biomarkers was constrained by the available data. Ideally, we would have examined biomarkers on the day of maximum AKI stage by functional criteria. Instead of the usual 48-hour window for the assessment of AKI, we chose the window from enrollment to 24 hours because it included the most patients with AKI, it had the maximum biomarker data available, and the timing of biomarkers measurement was closer to it. For biomarkers, we selected 6 hours after the beginning of resuscitation because previous studies reported that the [TIMP-2] × [IGFBP7] level is more informative when measured 6^34^ or 12 hours^35^ after initial therapy in patients with sepsis. Fourth, because of the exploratory and retrospective nature of our analysis, we could not determine sample sizes a priori and the study was likely underpowered for certain substages; we also could not fully adjust for multiple comparisons (multiple secondary end points and cutoffs). Our secondary end points included variables that were not adjusted for competing risk of death. Fifth, we did not have data on long-term kidney function (to calculate major adverse kidney events) and instead used 30-day survival as our main end point for evaluating the biomarker. Although we believe this end point is appropriate for septic shock, it would not be appropriate for conditions with low mortality (eg, after cardiac surgery). The use of more kidney-related secondary end points was motivated by an attempt to reduce this limitation.

## Conclusions

In this cohort study, integration of biomarkers of cell-cycle arrest in the staging process of AKI as proposed by ADQI-23 identified patients with different prognoses. Among patients who developed AKI within 24 hours after enrollment, a urinary [TIMP-2] × [IGFBP7] level greater than 2.0 (ng/mL)^2^/1000 was associated with greater mortality risk at 30 days when AKI stages were defined by kidney function. Our results suggest that in patients with septic shock who have developed AKI according to KDIGO staging, the addition of urinary [TIMP-2] × [IGFBP7] level may identify patients at any functional AKI stage who may have lower 30-day survival. Additional [TIMP-2] × [IGFBP7] cutoff levels explored in this study may deserve further investigation for the ability to stratify patients even in the absence of functional criteria for AKI.

## Notes

Supplement 1. **eFigure 1.** Study Flow **eFigure 2.** Covariate-adjusted Survival by New AKI Stages Based on [TIMP-2]•[IGFBP7]≤1.0 and >1.0 **eFigure 3.** Covariate-adjusted Survival Within Functional Stage Based on [TIMP-2]•[IGFBP7]≤1.0 and >1.0 **eFigure 4.** Covariate-adjusted Survival by New AKI Stages Based on [TIMP-2]•[IGFBP7]≤0.3 and >0.3 **eFigure 5.** Covariate-adjusted Survival Within Functional Stage Based on [TIMP-2]•[IGFBP7]≤0.3 and >0.3 **eTable 1.** General Characteristics of the Analysis and the Original Cohort **eTable 2.** General Characteristics According to the New AKI Stages Obtained Using [TIMP-2]•[IGFBP7]≤2.0 or >2.0 **eTable 3.** Secondary Endpoints Compared Between [TIMP-2]•[IGFBP7]≤1.0 and >1.0 Within the Same Functional KDIGO AKI Stage **eTable 4.** Secondary Endpoints Compared Between [TIMP-2]•[IGFBP7]≤0.3 and >0.3 Within the Same Functional KDIGO AKI Stage **eAppendix 1.** Missing Urinary [TIMP-2]•[IGFBP7] **eAppendix 2.** AKI Definition by KDIGO Criteria **eAppendix 3.** Sensitivity Analysis
eReferences

Supplement 2.
ProCESS and ProGReSS-AKI Investigators

## References

[1] Kellum JA, Lameire N, Aspelin P, et al. Kidney Disease: Improving Global Outcomes (KDIGO) Acute Kidney Injury Work Group: KDIGO clinical practice guideline for acute kidney injury. Kidney Int Suppl. 2012;2(1):1-138.

[2] Kudose S, Hoshi M, Jain S, Gaut JP. Renal histopathologic findings associated with severity of clinical acute kidney injury. *Am J Surg Pathol*. 2018;42(5):625-635. 10.1097/PAS.0000000000001028 29537990

[3] Chu R, Li C, Wang S, Zou W, Liu G, Yang L. Assessment of KDIGO definitions in patients with histopathologic evidence of acute renal disease. Clin J Am Soc Nephrol. 2014;9(7):1175-1182. doi:10.2215/CJN.06150613 24789552 PMC4078954

[4] Kidney Disease: Improving Global Outcomes (KDIGO) Chronic Kidney Disease Work Group. KDIGO clinical practice guideline for the evaluation and management of chronic kidney disease. Kidney Int Suppl. 2013;3:1-150.

[5] Kellum JA, Chawla LS. Cell-cycle arrest and acute kidney injury: the light and the dark sides. Nephrol Dial Transplant. 2016;31(1):16-22. doi:10.1093/ndt/gfv130 26044835 PMC4703048

[6] Kashani K, Cheungpasitporn W, Ronco C. Biomarkers of acute kidney injury: the pathway from discovery to clinical adoption. Clin Chem Lab Med. 2017;55(8):1074-1089. doi:10.1515/cclm-2016-0973 28076311

[7] Nickolas TL, Schmidt-Ott KM, Canetta P, . Diagnostic and prognostic stratification in the emergency department using urinary biomarkers of nephron damage: a multicenter prospective cohort study. J Am Coll Cardiol. 2012;59(3):246-255. doi:10.1016/j.jacc.2011.10.854 22240130 PMC3487165

[8] Bellomo R, Kellum JA, Ronco C, . Acute kidney injury in sepsis. Intensive Care Med. 2017;43(6):816-828. doi:10.1007/s00134-017-4755-7 28364303

[9] Srisawat N, Kellum JA. The role of biomarkers in acute kidney injury. Crit Care Clin. 2020;36(1):125-140. doi:10.1016/j.ccc.2019.08.010 31733675

[10] Ostermann M, Zarbock A, Goldstein S, . Recommendations on acute kidney injury biomarkers from the Acute Disease Quality Initiative Consensus Conference: a consensus statement. JAMA Netw Open. 2020;3(10):e2019209. doi:10.1001/jamanetworkopen.2020.19209 33021646

[11] Center for Devices and Radiological Health. Evaluation of automatic class III designation for NephroCheck® Test System: decision summary. US Food and Drug Administration. Accessed April 12, 2022. https://www.accessdata.fda.gov/cdrh_docs/reviews/DEN130031.pdf

[12] Emlet DR, Pastor-Soler N, Marciszyn A, . Insulin-like growth factor binding protein 7 and tissue inhibitor of metalloproteinases-2: differential expression and secretion in human kidney tubule cells. Am J Physiol Renal Physiol. 2017;312(2):F284-F296. doi:10.1152/ajprenal.00271.2016 28003188 PMC5336590

[13] Zarbock A, Schmidt C, Van Aken H, ; RenalRIPC Investigators. Effect of remote ischemic preconditioning on kidney injury among high-risk patients undergoing cardiac surgery: a randomized clinical trial. JAMA. 2015;313(21):2133-2141. doi:10.1001/jama.2015.4189 26024502

[14] Acute Disease Quality Initiative. ADQI 23 figures. Accessed April 12, 2022. https://www.adqi.org/images

[15] Joannidis M, Forni LG, Haase M, ; Sapphire Investigators. Use of cell cycle arrest biomarkers in conjunction with classical markers of acute kidney injury. *Crit Care Med*. 2019;47(10):e820-e826. 10.1097/CCM.0000000000003907 PMC6750148 31343478

[16] Xie Y, Ankawi G, Yang B, . Tissue inhibitor metalloproteinase-2 (TIMP-2) • IGF-binding protein-7 (IGFBP7) levels are associated with adverse outcomes in patients in the intensive care unit with acute kidney injury. Kidney Int. 2019;95(6):1486-1493. doi:10.1016/j.kint.2019.01.020 30982674

[17] Koyner JL, Shaw AD, Chawla LS, ; Sapphire Investigators. Tissue inhibitor metalloproteinase-2 (TIMP-2)•IGF-binding protein-7 (IGFBP7) levels are associated with adverse long-term outcomes in patients with AKI. J Am Soc Nephrol. 2015;26(7):1747-1754. doi:10.1681/ASN.2014060556 25535301 PMC4483589

[18] Husain-Syed F, Ferrari F, Sharma A, . Persistent decrease of renal functional reserve in patients after cardiac surgery-associated acute kidney injury despite clinical recovery. *Nephrol Dial Transplant*. 2019;34(2):308-317. 10.1093/ndt/gfy227 30053231

[19] Yealy DM, Kellum JA, Huang DT, ; ProCESS Investigators. A randomized trial of protocol-based care for early septic shock. N Engl J Med. 2014;370(18):1683-1693. doi:10.1056/NEJMoa1401602 24635773 PMC4101700

[20] Kellum JA, Chawla LS, Keener C, ; ProCESS and ProGReSS-AKI Investigators. The effects of alternative resuscitation strategies on acute kidney injury in patients with septic shock. Am J Respir Crit Care Med. 2016;193(3):281-287. doi:10.1164/rccm.201505-0995OC 26398704 PMC4803059

[21] World Medical Association. World Medical Association Declaration of Helsinki: ethical principles for medical research involving human subjects. JAMA. 2013;310(20):2191-2194. doi:10.1001/jama.2013.281053 24141714

[22] Kashani K, Al-Khafaji A, Ardiles T, . Discovery and validation of cell cycle arrest biomarkers in human acute kidney injury. Crit Care. 2013;17(1):R25. doi:10.1186/cc12503 23388612 PMC4057242

[23] Bihorac A, Chawla LS, Shaw AD, . Validation of cell-cycle arrest biomarkers for acute kidney injury using clinical adjudication. Am J Respir Crit Care Med. 2014;189(8):932-939. doi:10.1164/rccm.201401-0077OC 24559465

[24] Honore PM, Nguyen HB, Gong M, ; Sapphire and Topaz Investigators. Urinary tissue inhibitor of metalloproteinase-2 and insulin-like growth factor-binding protein 7 for risk stratification of acute kidney injury in patients with sepsis. Crit Care Med. 2016;44(10):1851-1860. doi:10.1097/CCM.0000000000001827 27355527 PMC5089124

[25] McCullough PA, Shaw AD, Haase M, . Diagnosis of acute kidney injury using functional and injury biomarkers: workgroup statements from the tenth Acute Dialysis Quality Initiative Consensus Conference. Contrib Nephrol. 2013;182:13-29. doi:10.1159/000349963 23689653

[26] Uhel F, Peters-Sengers H, Falahi F, ; MARS consortium. Mortality and host response aberrations associated with transient and persistent acute kidney injury in critically ill patients with sepsis: a prospective cohort study. Intensive Care Med. 2020;46(8):1576-1589. doi:10.1007/s00134-020-06119-x 32514599 PMC7381452

[27] Chawla LS, Bellomo R, Bihorac A, . Acute kidney disease and renal recovery: consensus report of the Acute Disease Quality Initiative (ADQI) 16 Workgroup. *Nat Rev Nephrol*. 2017;13(4):241-257. 10.1038/nrneph.2017.2 28239173

[28] Molinari L, Heskia F, Peerapornratana S, ; Sapphire and Protocolized Care for Early Septic Shock (ProCESS) Investigators. Limiting acute kidney injury progression in sepsis: study protocol and trial simulation. Crit Care Med. 2021;49(10):1706-1716. doi:10.1097/CCM.0000000000005061 33927121 PMC8439672

[29] Han WK, Wagener G, Zhu Y, Wang S, Lee HT. Urinary biomarkers in the early detection of acute kidney injury after cardiac surgery. Clin J Am Soc Nephrol. 2009;4(5):873-882. doi:10.2215/CJN.04810908 19406962 PMC2676184

[30] Vaidya VS, Ozer JS, Dieterle F, . Kidney injury molecule-1 outperforms traditional biomarkers of kidney injury in preclinical biomarker qualification studies. Nat Biotechnol. 2010;28(5):478-485. doi:10.1038/nbt.1623 20458318 PMC2885849

[31] Haase M, Devarajan P, Haase-Fielitz A, . The outcome of neutrophil gelatinase-associated lipocalin-positive subclinical acute kidney injury: a multicenter pooled analysis of prospective studies. J Am Coll Cardiol. 2011;57(17):1752-1761. doi:10.1016/j.jacc.2010.11.051 21511111 PMC4866647

[32] Albert C, Albert A, Kube J, . Urinary biomarkers may provide prognostic information for subclinical acute kidney injury after cardiac surgery. *J Thorac Cardiovasc Surg*. 2018;155(6):2441-2452.e13. 10.1016/j.jtcvs.2017.12.056 29366580

[33] Hoste EA, Bagshaw SM, Bellomo R, . Epidemiology of acute kidney injury in critically ill patients: the multinational AKI-EPI study. Intensive Care Med. 2015;41(8):1411-1423. doi:10.1007/s00134-015-3934-7 26162677

[34] Fiorentino M, Xu Z, Smith A, ; ProCESS and ProGReSS-AKI Investigators. Serial measurement of cell-cycle arrest biomarkers [TIMP-2]•[IGFBP7] and risk for progression to death, dialysis or severe acute kidney injury in patients with septic shock. Am J Respir Crit Care Med. Published online June 25, 2020. doi:10.1164/rccm.201906-1197OC 32584598 PMC7605192

[35] Nusshag C, Rupp C, Schmitt F, . Cell cycle biomarkers and soluble urokinase-type plasminogen activator receptor for the prediction of sepsis-induced acute kidney injury requiring renal replacement therapy: a prospective, exploratory study. Crit Care Med. 2019;47(12):e999-e1007. doi:10.1097/CCM.0000000000004042 31584458 PMC6867703

