# Supplementary material for: Utility of Biomarkers for Sepsis-Associated Acute Kidney Injury Staging
Source: JAMA Netw Open. 2022 May 18;5(5):e2212709. doi: 10.1001/jamanetworkopen.2022.12709 (PMC9118077; doi:10.1001/jamanetworkopen.2022.12709)
Supplement: Supplement 1. — eFigure 1. Study Flow eFigure 2. Covariate-adjusted Survival by New AKI Stages Based on [TIMP-2]•[IGFBP7]≤1.0 and >1.0 eFigure 3. Covariate-adjusted Survival Within Functional Stage Based on [TIMP-2]•[IGFBP7]≤1.0 and >1.0 eFigure 4. Covariate-adjusted Survival by New AKI Stages Based on [TIMP-2]•[IGFBP7]≤0.3 and >0.3 eFigure 5. Covariate-adjusted Survival Within Functional Stage Based on [TIMP-2]•[IGFBP7]≤0.3 and >0.3 eTable 1. General Characteristics of the Analysis and the Original Cohort eTable 2. General Characteristics According to the New AKI Stages Obtained Using [TIMP-2]•[IGFBP7]≤2.0 or >2.0 eTable 3. Secondary Endpoints Compared Between [TIMP-2]•[IGFBP7]≤1.0 and >1.0 Within the Same Functional KDIGO AKI Stage eTable 4. Secondary Endpoints Compared Between [TIMP-2]•[IGFBP7]≤0.3 and >0.3 Within the Same Functional KDIGO AKI Stage eAppendix 1. Missing Urinary [TIMP-2]•[IGFBP7] eAppendix 2. AKI Definition by KDIGO Criteria eAppendix 3. Sensitivity Analysis eReferences [file jamanetwopen-e2212709-s001.pdf]

Supplementary Online Content

Molinari L, Del Rio-Pertuz G, Smith A, et al; ProCESS and ProGReSS-AKI Investigators. Utility of biomarkers for sepsis-associated acute kidney injury staging. *JAMA Netw Open*. 2022;5(5):e2212709. doi:10.1001/jamanetworkopen.2022.12709

eFigure 1. Study Flow

eFigure 2. Covariate-adjusted Survival by New AKI Stages Based on [TIMP-2]•[IGFBP7] ≤1.0 and >1.0

eFigure 3. Covariate-adjusted Survival Within Functional Stage Based on [TIMP-2]•[IGFBP7] ≤1.0 and >1.0

eFigure 4. Covariate-adjusted Survival by New AKI Stages Based on [TIMP-2]•[IGFBP7] ≤0.3 and >0.3

eFigure 5. Covariate-adjusted Survival Within Functional Stage Based on [TIMP-2]•[IGFBP7] ≤0.3 and >0.3

eTable 1. General Characteristics of the Analysis and the Original Cohort

eTable 2. General Characteristics According to the New AKI Stages Obtained Using [TIMP-2]•[IGFBP7] ≤2.0 or >2.0

eTable 3. Secondary Endpoints Compared Between [TIMP-2]•[IGFBP7] ≤1.0 and >1.0 Within the Same Functional KDIGO AKI Stage

eTable 4. Secondary Endpoints Compared Between [TIMP-2]•[IGFBP7] ≤0.3 and >0.3 Within the Same Functional KDIGO AKI Stage

eAppendix 1. Missing Urinary [TIMP-2]•[IGFBP7]

eAppendix 2. AKI Definition by KDIGO Criteria

eAppendix 3. Sensitivity Analysis

eReferences

This supplementary material has been provided by the authors to give readers additional information about their work.

© 2022 Molinari L et al. *JAMA Network Open*.

SUPPLEMENTARY FIGURES

eFigure 1. Study flow

The figure shows how we obtained our analysis cohort.
ProCESS[1] was a multicenter, randomized clinical trial of three different resuscitation strategies in patients with septic shock that enrolled/randomized 1341 patients without finding any difference in mortality and need of organ support between the different strategies. Kellum and colleagues in a follow-up study[2] specifically evaluated if the presence and the severity of AKI were affected by the different resuscitation strategies. No association was found between different resuscitation strategies and development of AKI and its severity in a subgroup of 1243 patients resulted after excluding patients for whom the assessment of AKI was impossible or unreliable, such as patients with end-stage kidney disease (chronic dialysis not related to the current sepsis episode), reference sCr ≥4 mg/dL, or missing admission SCr. In our analysis, from the cohort of 1243 patients, we excluded patients with missing the measurements of urinary [TIMP-2]•[IGFBP7] at 6 hours from enrollment. The general characteristics of both our analysis cohort (999 patients) and the original cohort for the assessment of AKI from Kellum et al (1243 patients) are shown in eTable 1.

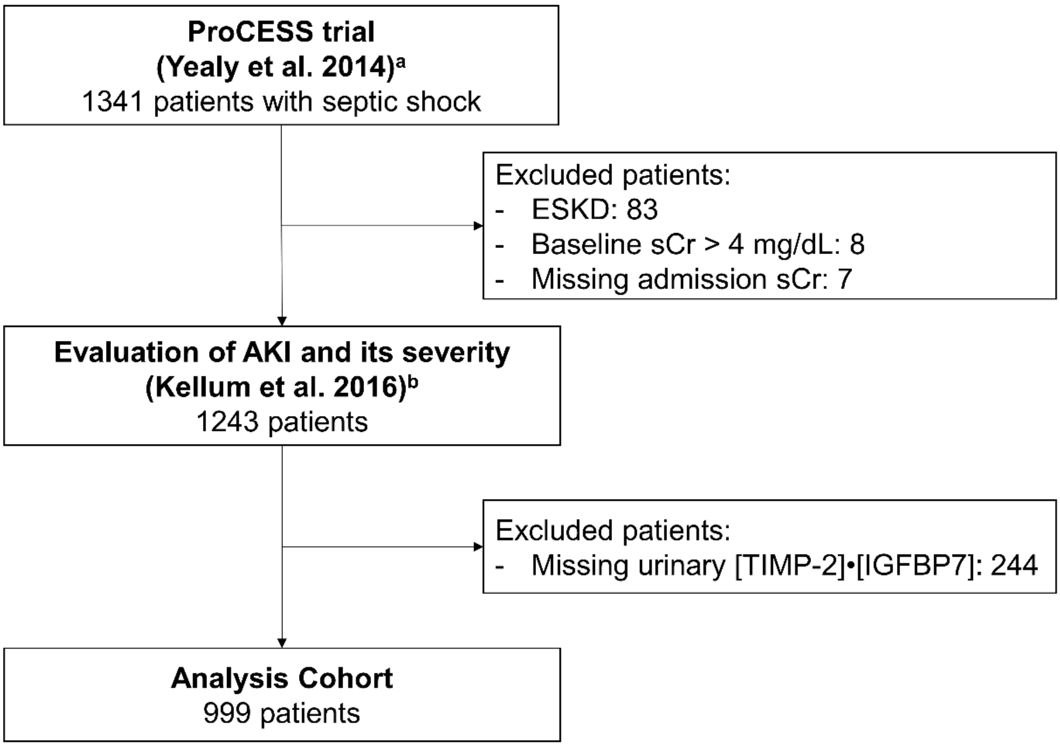

[a]Yealy DM, Kellum JA, Huang DT, et al. A randomized trial of protocol-based care for early septic shock. *N Engl J Med*. May 2014;370(18):1683-93. doi:10.1056/NEJMoa1401602
[b]Kellum JA, Chawla LS, Keener C, et al. The Effects of Alternative Resuscitation Strategies on Acute Kidney Injury in Patients with Septic Shock. *Am J Respir Crit Care Med*. Feb 2016;193(3):281-7. doi:10.1164/rccm.201505-0995OC

AKI = acute kidney injury; ESKD = end-stage kidney disease; IGFBP7 = insulin-like growth factor binding protein 7; sCr = serum creatinine; TIMP-2 = tissue inhibitor of metalloproteinases-2.

© 2022 Molinari L et al. *JAMA Network Open*.

eFigure 2. Covariate-adjusted survival by new AKI stages based on [TIMP-2]•[IGFBP7] ≤1.0 and >1.0.

Shown are the adjusted survival curves for the new AKI stages based on [TIMP-2]•[IGFBP7] ≤ or >1.0 (ng/mL)^2/1000. Dashed lines indicate biomarker negative patients and solid lines indicate those who were biomarker positive. Blue lines: No AKI/stage 1S; green: stage 1A/B; orange: stage 2A/B; red: stage 3A/B (for details about the definition of stages refer to Table 1). Covariates used in the Cox proportional hazard models were age, sex, race and Charlson Comorbidity Index. The Hazard Ratios of each stage are the following: No AKI: reference; Stage 1S: HR 2.20 (95%CI 1.11-4.34, *P*=.02); Stage 1A: HR 1.28 (95%CI 0.69-2.36, *P*=.43); Stage 1B: HR 2.39 (95%CI 1.14-5.02, *P*=.02); Stage 2A: HR 1.80 (95%CI 1.14-2.92, *P*=.02); Stage 2B: HR 3.60 (95%CI 2.28-5.68, *P*<.001); Stage 3A: HR 2.06 (95%CI 1.19-3.55, *P*=.01); Stage 3B: HR 3.68 (95%CI 2.21-6.15, *P*<.001). Numbers of patients at risk of death at the beginning of day 0, 10, 20 and 30 from enrollment are shown beneath each figure.

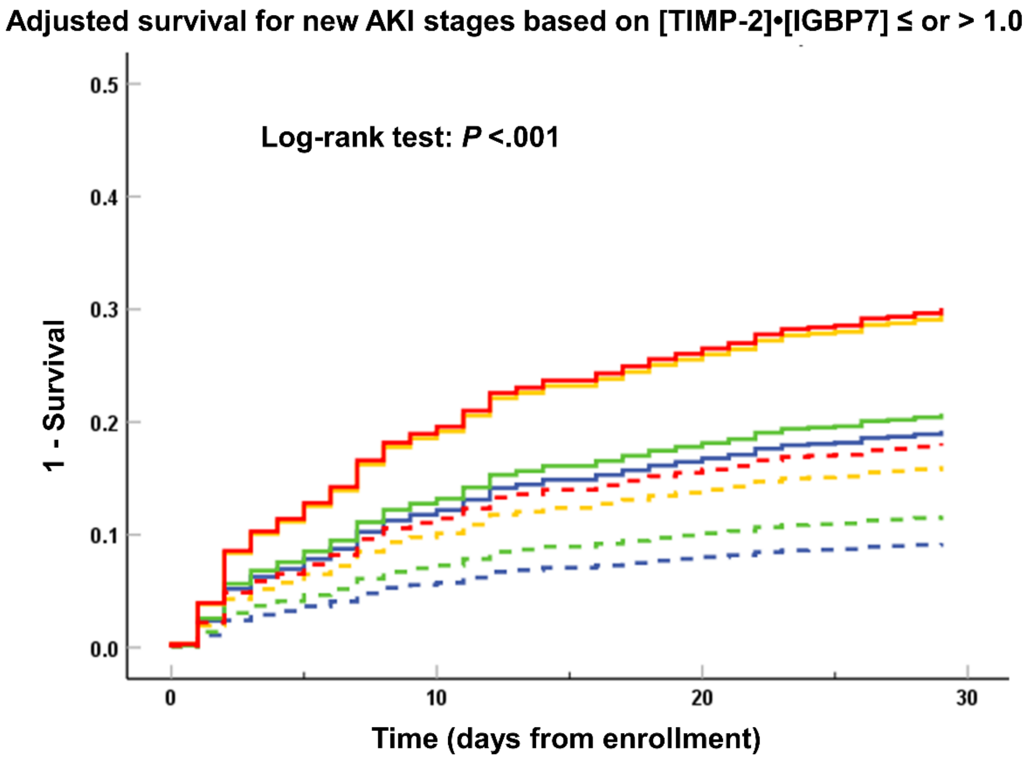

NUMBER AT RISK

	0	10	20	30
No AKI	321	306	295	287
Stage 1S	52	42	41	41
Stage 1A	116	108	105	101
Stage 1B	36	33	28	27
Stage 2A	173	152	142	140
Stage 2B	121	86	82	78
Stage 3A	98	86	80	77
Stage 3B	82	64	59	55

AKI = acute kidney injury; CI = confidence interval; HR = hazard ratio; IGFBP7 = insulin-like growth factor binding protein 7; TIMP-2 = tissue inhibitor of metalloproteinases-2.

© 2022 Molinari L et al. *JAMA Network Open*.

eFigure 3. **Covariate-adjusted survival within functional stage based on [TIMP-2]•[IGFBP7] ≤1.0 and >1.0.**

Each plot shows patients with the same KDIGO functional AKI stage (no AKI in panel A, stage 1 in panel B, stage 2 in panel C, and stage 3 in panel D) discriminating covariate-adjusted survival according to the presence of [TIMP-2]•[IGFBP7] ≤1.0 (dashed lines) or >1.0 (ng/mL)^2/1000 (solid lines). For details about the definition of stages refer to Table 1. Covariates used in the Cox proportional hazard models were age, sex, race and Charlson Comorbidity Index. Numbers of patients at risk of death at the beginning of day 0, 10, 20 and 30 from enrollment are shown beneath each figure.

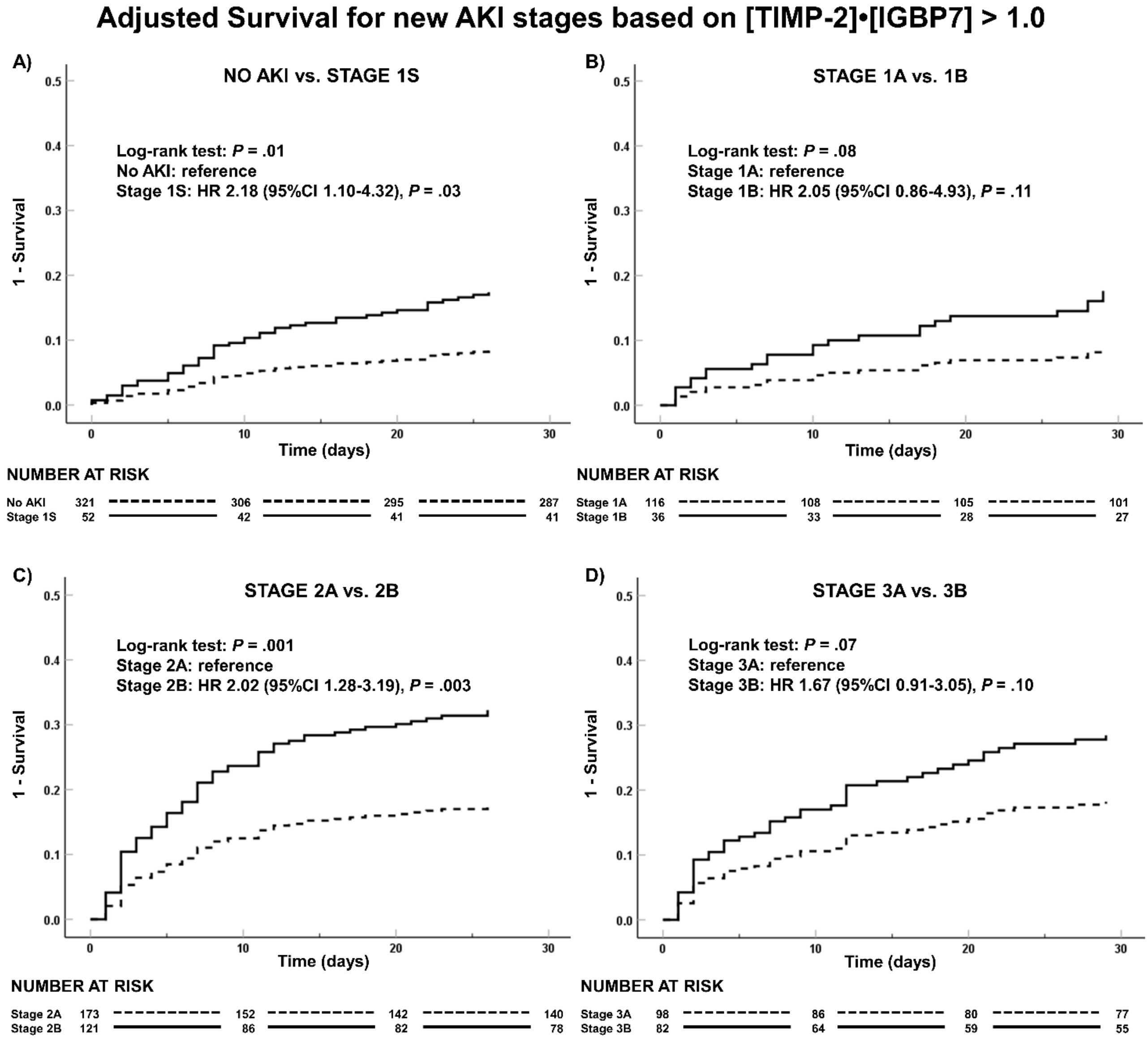

AKI = acute kidney injury; CI = confidence interval; HR = hazard ratio; IGFBP7 = insulin-like growth factor binding protein 7; TIMP-2 = tissue inhibitor of metalloproteinases-2.

© 2022 Molinari L et al. *JAMA Network Open*.

eFigure 4. Covariate-adjusted survival by new AKI stages based on [TIMP-2]•[IGFBP7] ≤0.3 and >0.3.

Shown are the adjusted survival curves for the new AKI stages based on [TIMP-2]•[IGFBP7] ≤ or >0.3 (ng/mL)^2/1000. Dashed lines indicate biomarker negative patients and solid lines indicate those who were biomarker positive. Blue lines: No AKI/stage 1S; green: stage 1A/B; orange: stage 2A/B; red: stage 3A/B (for details about the definition of stages refer to Table 1). Covariates used in the Cox proportional hazard models were age, sex, race and Charlson Comorbidity Index. The Hazard Ratios of each stage are the following: No AKI: reference; Stage 1S: HR 1.53 (95%CI 0.85-2.76, *P*=.16); Stage 1A: HR 0.88 (95%CI 0.34-2.31, *P*=.80); Stage 1B: HR 1.99 (95%CI 1.09-3.63, *P*=.03); Stage 2A: HR 1.61 (95%CI 0.88-2.92, *P*=.12); Stage 2B: HR 3.19 (95%CI 1.98-5.12, *P*<.001); Stage 3A: HR 2.06 (95%CI 1.03-4.11, *P*=.04); Stage 3B: HR 3.17 (95%CI 1.90-5.30, *P*<.001). Numbers of patients at risk of death at the beginning of day 0, 10, 20 and 30 from enrollment are shown beneath each figure.

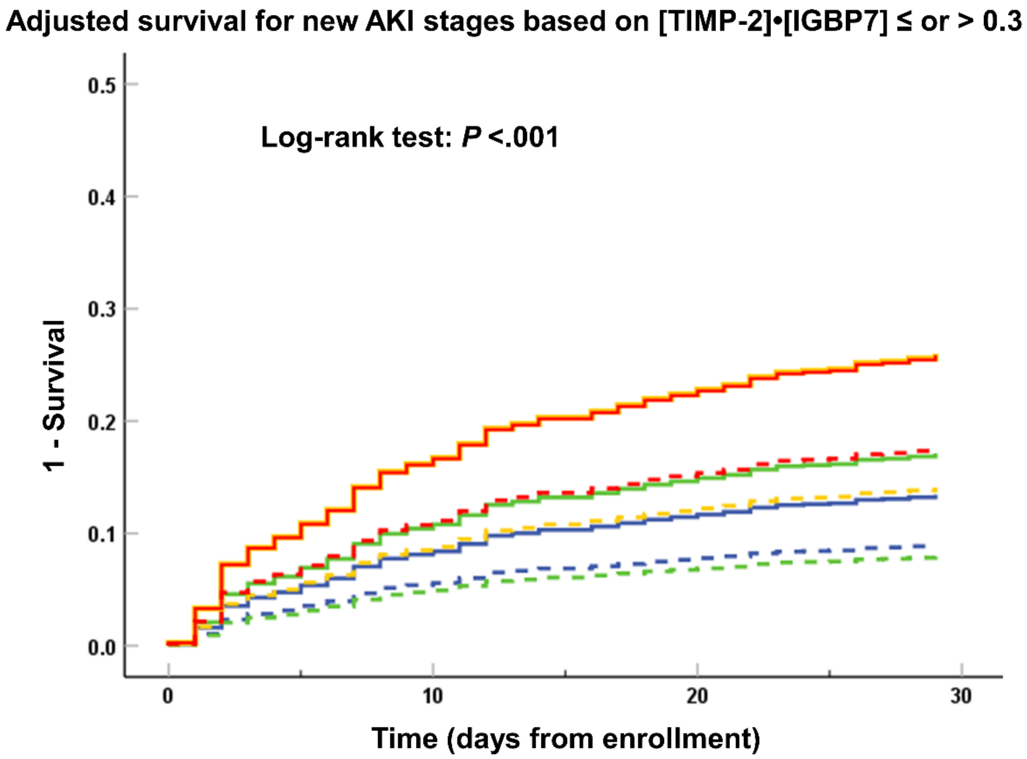

NUMBER AT RISK

No AKI	247	238	229	222
Stage 1S	126	110	107	106
Stage 1A	58	58	56	53
Stage 1B	94	85	77	75
Stage 2A	106	94	89	87
Stage 2B	188	144	135	131
Stage 3A	50	43	40	38
Stage 3B	130	107	99	94

AKI = acute kidney injury; CI = confidence interval; HR = hazard ratio; IGFBP7 = insulin-like growth factor binding protein 7; TIMP-2 = tissue inhibitor of metalloproteinases-2.

© 2022 Molinari L et al. *JAMA Network Open*.

eFigure 5. Covariate-adjusted survival within functional stage based on [TIMP-2]•[IGFBP7] ≤0.3 and >0.3.

Each plot shows patients with the same KDIGO functional AKI stage (no AKI in panel A, stage 1 in panel B, stage 2 in panel C, and stage 3 in panel D) discriminating covariate-adjusted survival according to the presence of [TIMP-2]•[IGFBP7] ≤ 0.3 (dashed lines) or >0.3 (ng/mL)[2]/1000 (solid lines). For details about the definition of stages refer to Table 1. Covariates used in the Cox proportional hazard models were age, sex, race and Charlson Comorbidity Index. Numbers of patients at risk of death at the beginning of day 0, 10, 20 and 30 from enrollment are shown beneath each figure.

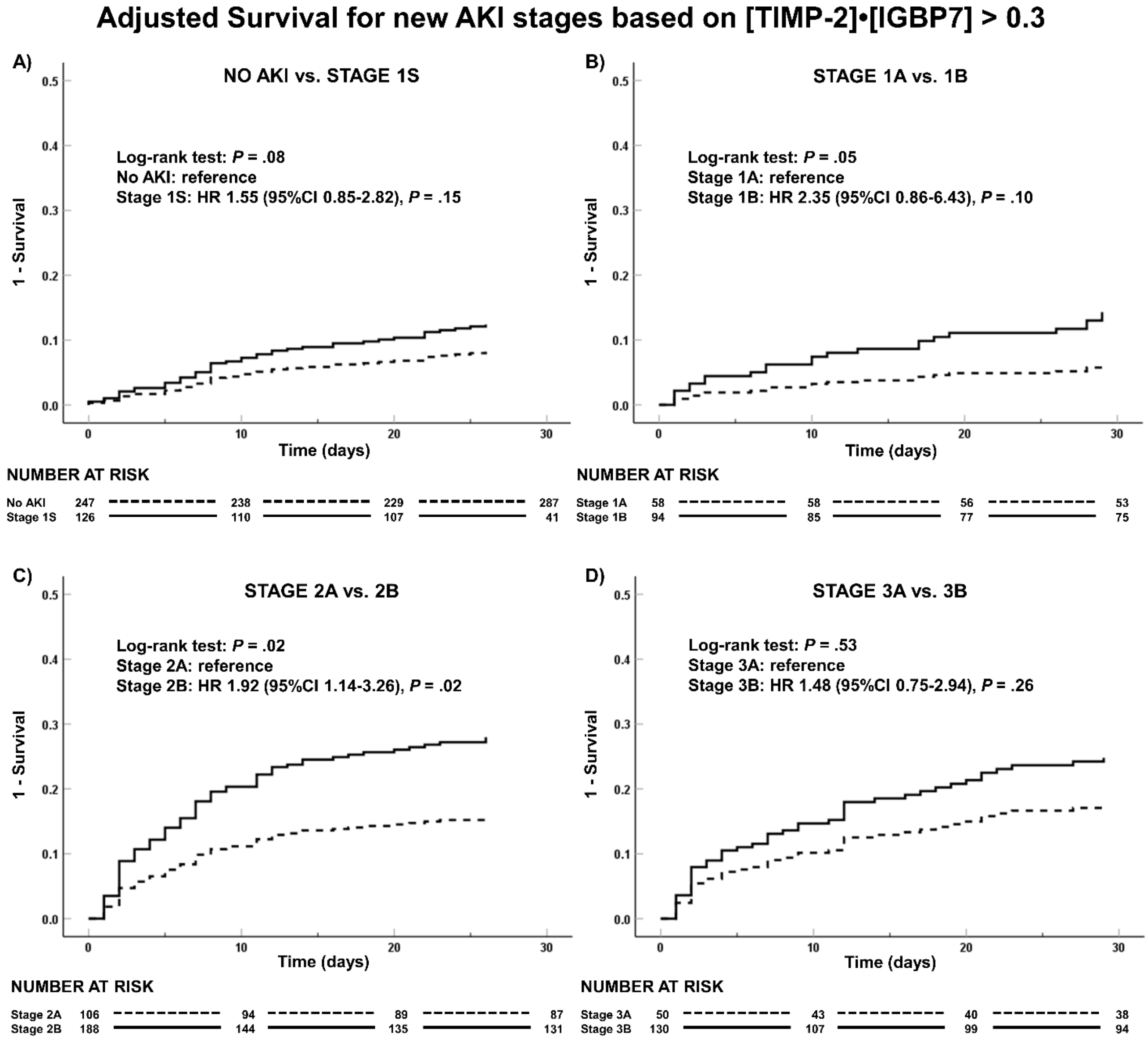

AKI = acute kidney injury; CI = confidence interval; HR = hazard ratio; IGFBP7 = insulin-like growth factor binding protein 7; TIMP-2 = tissue inhibitor of metalloproteinases-2.

© 2022 Molinari L et al. *JAMA Network Open*.

SUPPLEMENTARY TABLES

eTable 1. General characteristics of the analysis and the original cohort

The analysis cohort of 999 patients is a subset of the original cohort of 1243 patients as described in eFigure 1.

	Analysis cohort (N. 999)	Original cohort (N. 1243)
**Age, years**	61 [50-73]	61 [50-74]
**Sex, male/female**	554 (55.5%)/445 (44.5%)	688 (55.3%)/555 (44.7%)
**Race[a]**		
**African American/Black**	234 (23.4%)	289 (23.3%)
**Caucasian/White**	695 (69.6%)	870 (70.0%)
**Other**	70 (7.0%)	84 (6.8%)
**Cardiovascular disease[b]**	650 (65.1%)	806 (64.8%)
Arterial hypertension	584 (58.5%)	719 (57.8%)
Congestive heart failure	111 (11.1%)	140 (11.3%)
Previous myocardial infarction	108 (10.8%)	131 (10.5%)
Cerebral vascular disease	105 (10.5%)	119 (9.6%)
Peripheral vascular disease	75 (7.5%)	92 (7.4%)
**Diabetes mellitus**	333 (33.3%)	406 (32.7%)
**Chronic respiratory disease**	234 (23.4%)	281 (22.6%)
**Renal disease history**	101 (10.1%)	127 (10.2%)
**Active cancer**	179 (17.9%)	227 (18.3%)
**Dementia**	78 (7.8%)	98 (7.9%)
**Liver cirrhosis**	59 (5.9%)	79 (6.4%)
**Peptic ulcer disease**	54 (5.4%)	68 (5.5%)
**HIV infection**	25 (2.5%)	34 (2.7%)
**Charlson comorbidity index**	2 [1-4]	2 [1-4]
**SOFA score at enrollment**	7 [4-9]	7 [4-9]
**APACHE II score at enrollment**	19 [15-24]	19 [15-25]
**AKI (0-24 hours from enrollment)**	629 (62.7%)	801 (64.4%)
no AKI	373 (37.3%)	442 (35.6%)
KDIGO Stage 1	152 (15.2%)	194 (15.6%)
KDIGO Stage 2	294 (29.4%)	377 (30.3%)
KDIGO Stage 3	180 (18.0%)	230 (18.5%)
**Mortality at 30 days**	193 (19.3%)	281 (22.6%)
**Hospital LOS, days**	8 [5-14]	8 [5-14]
**[TIMP-2]•[IGFBP7] at 6 hours, $(ng/mL)^2/1000$**	0.35 [0.12-1.41]	0.35 [0.12-1.41][c]
**[TIMP-2]•[IGFBP7] >2.0 $(ng/mL)^2/1000$**	196 (19.6%)	196 (19.6%)[c]
**[TIMP-2]•[IGFBP7] >1.0 $(ng/mL)^2/1000$**	291 (29.1%)	291 (29.1%)[c]
**[TIMP-2]•[IGFBP7] >0.3 $(ng/mL)^2/1000$**	538 (53.9%)	538 (53.9%)[c]

© 2022 Molinari L et al. *JAMA Network Open*.

There are no statistically significant differences between the two cohorts for all the variables reported (*P*>.05). Categorical variables are presented as numbers (%), continuous variables as medians [interquartile range].
[a]Race was determined by patient self-report or by patient's legally authorized representative. Other race corresponds to Asian, American Indian or native Alaskan, Native Hawaiian or other Pacific islander, unknown, or other.
[b]Presence of any among arterial hypertension, congestive heart failure, previous myocardial infarction, cerebral vascular disease, and peripheral vascular disease.
[c]These values are the same values of the left column since the biomarkers were measured only in the 999 patients who constitute the analysis cohort (244 missing values).

AKI = acute kidney injury; APACHE = acute physiology and chronic health evaluation; IGFBP7 = insulin-like growth factor binding protein 7; KDIGO = Kidney Disease: Improving Global Outcomes; LOS = length of stay; SOFA = sequential organ failure assessment; TIMP-2 = tissue inhibitor of metalloproteinases-2.

© 2022 Molinari L et al. *JAMA Network Open*.

eTable 2. General characteristics according to the new AKI stages obtained using [TIMP-2]•[IGFBP7] ≤2.0 or >2.0.
The stages are defined according to the criteria presented in Table 1.

	**No AKI (N. 344)**	**Stage 1S (N. 29)**	**Stage 1A (N. 128)**	**Stage 1B (N. 24)**	**Stage 2A (N. 213)**	**Stage 2B (N. 81)**	**Stage 3A (N. 118)**	**Stage 3B (N. 62)**
**Age, years**	59 [48-71]	59 [47-65]	62 [53-75]	65 [57-81]	62 [50-76]	67 [57-77]	59 [50-73]	61 [53-73]
**Sex, male/female**	157 (45.6%) / 187 (54.4%)	15 (51.7%) / 14 (48.3%)	87 (68%) / 41 (32%)	18 (75%) / 6 (25%)	119 (55.9%) / 94 (44.1%)	50 (61.7%) / 31 (38.3%)	61 (51.7%) / 7 (48.3%)	47 (75.8%) / 15 (24.2%)
**Race[a]**								
**African American/Black**	64 (18.6%)	6 (20.7%)	30 (23.4%)	2 (8.3%)	52 (24.4%)	23 (28.4%)	40 (33.9%)	17 (27.4%)
**Caucasian/White**	253 (73.5%)	18 (62.1%)	93 (72.7%)	19 (79.2%)	145 (68.1%)	55 (67.9%)	70 (59.3%)	42 (67.7%)
**Other**	27 (7.8%)	5 (17.2%)	5 (3.9%)	3 (12.5%)	16 (7.5%)	3 (3.7%)	8 (6.8%)	3 (4.8%)
**Cardiovascular disease[b]**	204 (59.3%)	20 (69%)	80 (62.5%)	16 (66.7%)	146 (68.5%)	59 (72.8%)	85 (72%)	40 (64.5%)
Arterial hypertension	181 (52.6%)	16 (55.2%)	73 (57%)	15 (62.5%)	132 (62%)	53 (65.4%)	79 (66.9%)	35 (56.5%)
Congestive heart failure	34 (9.9%)	4 (13.8%)	15 (11.7%)	3 (12.5%)	22 (10.3%)	7 (8.6%)	21 (17.8%)	5 (8.1%)
Previous myocardial infarction	37 (10.8%)	3 (10.3%)	15 (11.7%)	4 (16.7%)	20 (9.4%)	8 (9.9%)	15 (12.7%)	6 (9.7%)
Cerebral vascular disease	26 (7.6%)	1 (3.4%)	10 (7.8%)	2 (8.3%)	33 (15.5%)	11 (13.6%)	11 (9.3%)	11 (17.7%)
Peripheral vascular disease	24 (7%)	2 (6.9%)	10 (7.8%)	1 (4.2%)	24 (11.3%)	8 (9.9%)	4 (3.4%)	2 (3.2%)
**Diabetes mellitus**	104 (30.2%)	7 (24.1%)	39 (30.5%)	8 (33.3%)	77 (36.2%)	30 (37%)	46 (39%)	22 (35.5%)
**Chronic respiratory disease**	82 (23.8%)	4 (13.8%)	28 (21.9%)	10 (41.7%)	49 (23%)	11 (13.6%)	42 (35.6%)	8 (12.9%)
**Renal disease history**	17 (4.9%)	3 (10.3%)	6 (4.7%)	1 (4.2%)	23 (10.8%)	12 (14.8%)	21 (17.8%)	18 (29%)
**Active cancer**	62 (18%)	7 (24.1%)	19 (14.8%)	7 (29.2%)	36 (16.9%)	21 (25.9%)	14 (11.9%)	13 (21%)
**Dementia**	26 (7.6%)	1 (3.4%)	13 (10.2%)	3 (12.5%)	15 (7%)	6 (7.4%)	9 (7.6%)	5 (8.1%)
**Liver cirrhosis**	14 (4.1%)	4 (13.8%)	6 (4.7%)	1 (4.2%)	20 (9.4%)	3 (3.7%)	5 (4.2%)	6 (9.7%)
**Peptic ulcer disease**	14 (4.1%)	4 (13.8%)	6 (4.7%)	1 (4.2%)	14 (6.6%)	2 (2.5%)	9 (7.6%)	4 (6.5%)
**HIV infection**	11 (3.2%)	0 (0%)	1 (0.8%)	0 (0%)	9 (4.2%)	0 (0%)	3 (2.5%)	1 (1.6%)
**Charlson comorbidity index**	2 [1-3]	1 [1-4]	1 [0-3]	2 [1-4]	2 [1-4]	2 [1-4]	2 [1-4]	3 [1-4]
**SOFA score at enrollment**	5 [3-7]	7 [5-9]	6 [4-9]	7 [5-11]	7 [5-9]	8 [7-11]	9 [6-11]	10 [7-12]
**APACHE II score at enrollment**	17 [13-21]	19 [13-23]	18 [14-21]	21 [17-26]	20 [16-25]	22 [18-25]	25 [20-29]	25 [21-30]

Categorical variables are reported as numbers (%), continuous variables as medians [interquartile range].

© 2022 Molinari L et al. *JAMA Network Open*.

[a]Race was determined by patient self-report or by patient’s legally authorized representative. Other race corresponds to Asian, American Indian or native Alaskan, Native Hawaiian or other Pacific islander, unknown, or other.
[b]Presence of any among arterial hypertension, congestive heart failure, previous myocardial infarction, cerebral vascular disease, and peripheral vascular disease.

APACHE = acute physiology and chronic health evaluation; IGFBP7 = insulin-like growth factor binding protein 7; SOFA = sequential organ failure assessment; TIMP-2 = tissue inhibitor of metalloproteinases-2.

© 2022 Molinari L et al. *JAMA Network Open*.

eTable 3. Secondary endpoints compared between [TIMP-2]•[IGFBP7] ≤1.0 and >1.0 within the same functional KDIGO AKI stage.

The second column shows the values of the endpoints for each KDIGO functional stage (no AKI, stage 1, stage 2 and stage 3), the third and fourth columns compare the endpoints according to the new AKI stages that separate each KDIGO stage according to the presence of [TIMP-2]•[IGFBP7] ≤1.0 or >1.0. The last columns show the measures of effect and *P* value of the statistical comparison between the third and fourth column.

	**KDIGO stage**	**[TIMP-2]•[IGFBP7] ≤1.0**	**[TIMP-2]•[IGFBP7] >1.0**	**Relative Risk or Median Difference[a] (95% CI)**	***P*[b]**
	**no AKI (N. 373)**	**No AKI (N. 321)**	**Stage 1S (N. 52)**		
**Mortality at 30 days**	12.1% (9.1, 15.7)	10.6% (7.6, 14.3)	21.2% (11.8, 33.6)	2.00 (1.08, 3.69)	.03
**Full recovery**	-	-	-	-	-
**AKI stage 3 by day 7**	4.0% (2.4, 6.4)	4.0% (2.3, 6.6)	3.8% (0.8, 11.8)	0.95 (0.22, 4.09)	.95
**Hospital LOS, days**	7 [5-12]	7 [4-10]	11 [7-22]	4 (2, 6)	<.001
	**Stage 1 (N. 152)**	**Stage 1A (N. 116)**	**Stage 1B (N. 36)**		
**Mortality at 30 days**	15.8% (10.7, 22.2)	12.9% (7.8, 19.9)	25.0% (13.2, 40.7)	1.93 (0.93, 4.04)	.08
**Full recovery**	80.3% (73.4, 86)	82.8% (75.1, 88.8)	72.2% (56.3, 84.7)	0.87 (0.70, 1.09)	.17
**AKI stage 3 by day 7**	5.3% (2.5, 9.7)	4.3% (1.7, 9.2)	8.3% (2.4, 20.6)	1.93 (0.49, 7.70)	.35
**Hospital LOS, days**	9 [6-13]	9 [6-13]	8 [6-12]	0 (-3, 2)	.89
	**Stage 2 (N. 294)**	**Stage 2A (N. 173)**	**Stage 2B (N. 121)**		
**Mortality at 30 days**	25.9% (21.1, 31.1)	19.1% (13.8, 25.4)	35.5% (27.4, 44.3)	1.86 (1.26, 2.75)	.002
**Full recovery**	65% (59.4, 70.3)	72.8% (65.9, 79)	53.7% (44.8, 62.4)	0.74 (0.61, 0.89)	.001
**AKI stage 3 by day 7**	19.4% (15.2, 24.2)	13.9% (9.3, 19.6)	27.3% (19.9, 35.7)	1.97 (1.23, 3.15)	.004
**Hospital LOS, days**	10 [6-15]	9 [6-14]	11 [6-17]	1 (0, 3)	.08
	**Stage 3 (N. 180)**	**Stage 3A (N. 98)**	**Stage 3B (N. 82)**		
**Mortality at 30 days**	26.7% (20.6, 33.5)	21.4% (14.2, 30.3)	32.9% (23.5, 43.6)	1.54 (0.94, 2.51)	.08
**Full recovery**	47.8% (40.6, 55.1)	56.1% (46.2, 65.7)	37.8% (27.9, 48.6)	0.67 (0.49, 0.94)	.01
**AKI stage 3 by day 7**	-	-	-	-	-
**Hospital LOS, days**	11 [7-20]	11 [6-18]	10 [7-22]	1 (-1, 4)	.47

Categorical variables are presented as percentages (95%CI lower limit, 95%CI upper limit), continuous variables as medians [25th percentile-75th percentile], relative risks and median differences as numbers (95%CI lower limit, 95%CI upper limit).
[a]Independent-Samples Hodges-Lehman Median Difference
[b]*P* value refers to Chi-Square test or Mann-Whitney U test used as appropriate.

AKI = acute kidney injury; CI = confidence interval; IGFBP7 = insulin-like growth factor binding protein 7; KDIGO = Kidney Disease: Improving Global Outcomes; LOS = length of stay; TIMP-2 = tissue inhibitor of metalloproteinases-2.

© 2022 Molinari L et al. *JAMA Network Open*.

eTable 4. Secondary endpoints compared between [TIMP-2]•[IGFBP7] ≤0.3 and >0.3 within the same functional KDIGO AKI stage.

The second column shows the values of the endpoints for each KDIGO functional stage (no AKI, stage 1, stage 2 and stage 3), the third and fourth columns compare the endpoints according to the new AKI stages that separate each KDIGO stage according to the presence of [TIMP-2]•[IGFBP7] ≤0.3 or >0.3. The last columns show the measures of effect and *P* value of the statistical comparison between the third and fourth column.

	**KDIGO stage**	**[TIMP-2]•[IGFBP7] ≤0.3**	**[TIMP-2]•[IGFBP7] >0.3**	**Relative Risk or Median Difference[a] (95% CI)**	***P*[b]**
	**no AKI (N. 373)**	**No AKI (N. 247)**	**Stage 1S (N. 126)**		
**Mortality at 30 days**	12.1% (9.1, 15.7)	10.1% (6.8, 14.3)	15.9% (10.3, 23)	1.57 (0.91, 2.71)	.11
**Full recovery**	-	-	-	-	-
**AKI stage 3 by day 7**	4.0% (2.4, 6.4)	4.5% (2.4, 7.6)	3.2% (1.1, 7.4)	0.71 (0.23, 2.19)	.55
**Hospital LOS, days**	7 [5-12]	7 [4-10]	9 [6-14]	2 (1, 4)	<.001
	**Stage 1 (N. 152)**	**Stage 1A (N. 58)**	**Stage 1B (N. 94)**	**Relative Risk (95% CI)**	
**Mortality at 30 days**	15.8% (10.7, 22.2)	8.6% (3.4, 17.9)	20.2% (13.1, 29.2)	2.35 (0.93, 5.94)	.06
**Full recovery**	80.3% (73.4, 86)	86.2% (75.7, 93.3)	76.6% (67.3, 84.3)	0.89 (0.76, 1.03)	.15
**AKI stage 3 by day 7**	5.3% (2.5, 9.7)	3.4% (0.7, 10.6)	6.4% (2.7, 12.7)	1.85 (0.39, 8.87)	.43
**Hospital LOS, days**	9 [6-13]	8 [6-11]	10 [6-16]	1 (0, 3)	.11
	**Stage 2 (N. 294)**	**Stage 2A (N. 106)**	**Stage 2B (N. 188)**	**Relative Risk (95% CI)**	
**Mortality at 30 days**	25.9% (21.1, 31.1)	17.9% (11.5, 26)	30.3% (24.1, 37.2)	1.69 (1.07, 2.68)	.02
**Full recovery**	65% (59.4, 70.3)	78.3% (69.8, 85.3)	57.4% (50.3, 64.4)	0.73 (0.62, 0.86)	<.001
**AKI stage 3 by day 7**	19.4% (15.2, 24.2)	11.3% (6.3, 18.4)	23.9% (18.3, 30.4)	2.11 (1.17, 3.82)	.009
**Hospital LOS, days**	10 [6-15]	8 [6-13]	11 [7-16]	2 (0, 3)	.03
	**Stage 3 (N. 180)**	**Stage 3A (N. 50)**	**Stage 3B (N. 130)**		
**Mortality at 30 days**	26.7% (20.6, 33.5)	24% (13.8, 37.1)	27.7% (20.6, 35.8)	1.15 (0.66, 2.03)	.62
**Full recovery**	47.8% (40.6, 55.1)	62% (48.2, 74.5)	42.3% (34.1, 50.9)	0.68 (0.51, 0.92)	.02
**AKI stage 3 by day 7**	-	-	-	-	-
**Hospital LOS, days**	11 [7-20]	9 [5-13]	11 [7-21]	2 (-1, 5)	.16

Categorical variables are presented as percentages (95%CI lower limit, 95%CI upper limit), continuous variables as medians [25th percentile-75th percentile], relative risks and median differences as numbers (95%CI lower limit, 95%CI upper limit).
[a]Independent-Samples Hodges-Lehman Median Difference
[b]*P* value refers to Chi-Square test or Mann-Whitney U test used as appropriate.

AKI = acute kidney injury; CI = confidence interval; IGFBP7 = insulin-like growth factor binding protein 7; KDIGO = Kidney Disease: Improving Global Outcomes; LOS = length of stay; TIMP-2 = tissue inhibitor of metalloproteinases-2.

© 2022 Molinari L et al. *JAMA Network Open*.

eAppendix 1. MISSING URINARY [TIMP-2]•[IGFBP7]

The two biomarkers were measured after the end of the trial, randomly selecting 1000 urine samples for the timepoint at 6 hours from enrollment among the available and stored samples for the entire ProCESS trial (that consisted of 1341 patients). The samples were centrifuged right after collection, and the supernatant was frozen and stored at <-70°C. The supernatant was then thawed immediately prior to testing for two biomarkers, tissue inhibitor of metalloproneises-2 (TIMP-2) and insulin-like growth factor binding protein 7 (IGFBP7) with the clinical immunoassay NephroCheck® Test (Astute Medical, San Diego, CA, USA) performed according to the manufacturer's specifications.

It is possible that the random selection of the urine sample of some patients was not possible because their urine samples were not collected and/or unavailable for some of the following reasons:

- patients with septic shock could have been severely ill with severe oliguria or anuria, thus preventing the collection of urine samples. But only around 6% (15/244) of the patients missing [TIMP-2]•[IGFBP7] at 6 hours from enrollment had AKI stage 3 using KDIGO criteria for urine output.
- patients with septic shock could have died in the first hours from enrollment, preventing the collection of urine samples. However, only 6% (15/244) of the patients with missing [TIMP-2]•[IGFBP7] at 6 hours from enrollment died in the same day of enrollment. Unfortunately, the exact hour of death was not available in our dataset, and we cannot assess if death occurred within the first 6 hours.
- for the original purposes of the original ProCESS trial, collection of urine samples was not a mandatory condition, so it could be possible that other contingent situations could have prevented the site personnel from collecting the urine samples. Unfortunately, this condition was not reported in our dataset and we cannot determine how many patients experienced that.

© 2022 Molinari L et al. *JAMA Network Open*.

- the patient did not give the consent to perform tests on his/her biological samples or, after initial consent, he/she withdrew from the study preventing further collection/use of samples and/or data. Around 4% (10/244) of the patients missing [TIMP-2]•[IGFBP7] at 6 hours from enrollment underwent one of these conditions.

© 2022 Molinari L et al. *JAMA Network Open*.

eAppendix 2. AKI DEFINITION BY KDIGO CRITERIA

Methods

We used both serum creatinine (sCr) and urine output (UO) Kidney Disease: Improving Global Outcomes (KDIGO)[3] criteria to define Acute Kidney Injury (AKI) stage. As recommended by the guidelines, we used the worst (highest) stage between the two criteria to define the AKI stage. The detailed methods by which AKI was assessed in our analysis was reported in the first follow-up study of the ProCESS trial published by Kellum and colleagues.[2] Briefly, the presence (or absence) of AKI at enrollment (0 hours) was based only on sCr at the time of enrollment comparing it with the reference creatinine value. Reference creatinine was the lowest value of sCr between baseline (preadmission, the most recent pre-hospital value up to 1-year prior index event) and admission (recorded in the first 24 hours of hospital admission). The stage of AKI was determined each day based on maximum severity by either sCr or UO criteria, but UO was recorded only until ICU discharge or 72 hours. AKI stage by OU criteria was assigned only if the urine output values were recorded. If UO was missing, no imputation was done, and it did not contribute to staging for patients.

Results

Regarding our main exposure, the presence of AKI between 0-24 hours from enrollment, the results were as follows:

A. 403/626 patients (64.4%) had the highest AKI stage already at enrollment (0 hours), and it was defined using serum creatinine criteria;
B. 223/626 patients (35.6%) had their highest AKI within the first day:
    a. For 216 patients (out of 223), AKI highest stage was defined using both sCr and UO KDIGO criteria:
        i. for 51, AKI stage was defined by sCr criteria;

© 2022 Molinari L et al. *JAMA Network Open*.

ii. 16 patients had the same stage for sCr and UO criteria;

iii. for 149 patients, AKI stage was defined by UO criteria.

b. For 7 patients, urine output data were missing, and only sCr criteria were used.

© 2022 Molinari L et al. *JAMA Network Open*.

eAppendix 3. SENSITIVITY ANALYSIS

Methods

In this sensitivity analysis, we also evaluated the potential roles of 0.3 and 1.0 cutoffs for [TIMP-2]•[IGFBP7]. The 0.3 cutoff, which is considered a “high-sensitivity cutoff” from the results both in its derivation[4] and validation studies,[5] and the 1.0 cutoff, which has already been evaluated in sepsis and showed good sensitivity and specificity compared to 0.3 and 2.0 cutoffs.[6] We used these cutoffs to categorize patients according to the new AKI staging system proposed by ADQI-23 (Table 1) and we conducted a similar analysis to what was described and performed for the 2.0 cutoff in the main paper.

Results - [TIMP-2]•[IGFBP7] >1.0

A total of 291/999 (29.1%) patients had [TIMP-2]•[IGFBP7]>1.0 $(ng/mL)^2/1000$. We applied the 1.0 cutoff of [TIMP-2]•[IGFBP7] to each functional KDIGO AKI stage. For [TIMP-2]•[IGFBP7], among patients with no AKI, 52/373 (13.9%) had [TIMP-2]•[IGFBP7]>1.0 (stage 1S). Among patients with AKI stage 1, 36/152 (23.7%) had [TIMP-2]•[IGFBP7]>1.0 (stage 1B). For AKI stage 2, 121/294 (41.2%) had [TIMP-2]•[IGFBP7]>1.0 (stage 2B) and for AKI stage 3, 82/180 (45.6%) had [TIMP-2]•[IGFBP7]>1.0 (stage 3B). The survival was different across all 8 stages obtained with [TIMP-2]•[IGFBP7] (*P*<.001) and the corresponding covariate-adjusted survival curves are shown in eFigure 2 and the hazard ratios (HR) for each stage are reported in the corresponding legend. The pairwise comparison of survival within the same functional stage using Log-rank test was statistically different for No AKI vs. stage 1S (*P*=.01, eFigure 3A) and stage 2A vs. 2B (*P*=.001, eFigure 3C). On the contrary, the pairwise comparisons of survival for stage 1A vs. 1B (*P*=.08, eFigure 3B) and stage 3A vs. 3B (*P*=.07, eFigure 3D) were not different. Similarly, a significantly increased HR was obtained when comparing stage 1S to No AKI with HR 2.18 (95%CI 1.10-4.32, *P*=.03, eFigure 3A) and stage 2B to 2A with HR 2.02

© 2022 Molinari L et al. *JAMA Network Open*.

(95%CI 1.28-3.19, *P*=.003, eFigure 3C). The HR for stage 1B compared to 1A was 2.05 (95%CI 0.86-4.93, *P*=.11, eFigure 3B) and for stage 3B compared to 3A was 1.67 (95%CI 0.91-3.05, *P*=.10, eFigure 3D). eTable 3 summarizes the secondary endpoints according to each KDIGO stage and then compares them within each stage between [TIMP-2]•[IGFBP7]≤1.0 and >1.0.

Results - [TIMP-2]•[IGFBP7] >0.3

A total of 538/999 (53.9%) patients had [TIMP-2]•[IGFBP7]>0.3 $(ng/mL)^2/1000$. We applied the 0.3 cutoff of [TIMP-2]•[IGFBP7] to each functional KDIGO AKI stage. For [TIMP-2]•[IGFBP7], among patients with no AKI, 126/373 (33.8 %) had [TIMP-2]•[IGFBP7]>0.3 (stage 1S). Among patients with AKI stage 1, 94/152 (61.8%) had [TIMP-2]•[IGFBP7]>0.3 (stage 1B). For AKI stage 2, 188/294 (63.9%) had [TIMP-2]•[IGFBP7]>0.3 (stage 2B) and for AKI stage 3, 130/180 (72.2%) had [TIMP-2]•[IGFBP7]>0.3 (stage 3B). The survival was different across all 8 stages obtained with [TIMP-2]•[IGFBP7] (*P*<.001) and the corresponding covariate-adjusted survival curves are shown in eFigure 4 and HR for each stage are reported in the corresponding legend. The pairwise comparison of survival within the same functional stage using Log-rank test was statistically different only for stage 2A vs. 2B (*P*=.02, eFigure 5C). On the contrary, the pairwise comparisons of survival for No AKI vs. stage 1S (*P*=.08, eFigure 5A), stage 1A vs. 1B (*P*=.05, eFigure 5B) and stage 3A vs. 3B (*P*=.53, eFigure 5D) were not statistically different. Similarly, a significantly increased HR was obtained only when comparing stage 2B to 2A with a HR 1.92 (95%CI 1.14-3.26, *P*=.02, eFigure 5C). eTable 4 summarizes the secondary endpoints according to each KDIGO stage and then comparing them within each stage between [TIMP-2]•[IGFBP7]≤1.0 and >1.0.

© 2022 Molinari L et al. *JAMA Network Open*.

eReferences

1. Yealy DM, Kellum JA, Huang DT, et al. A randomized trial of protocol-based care for early septic shock. *N Engl J Med*. May 2014;370(18):1683-93. doi:10.1056/NEJMoa1401602
2. Kellum JA, Chawla LS, Keener C, et al. The Effects of Alternative Resuscitation Strategies on Acute Kidney Injury in Patients with Septic Shock. *Am J Respir Crit Care Med*. Feb 2016;193(3):281-7. doi:10.1164/rccm.201505-0995OC
3. Kidney Disease: Improving Global Outcomes (KDIGO) Acute Kidney Injury Work Group. KDIGO Clinical Practice Guideline for Acute Kidney Injury. *Kidney Int Suppl*. 2012;2(1):1-138. doi:10.1038/kisup.2012.1
4. Kashani K, Al-Khafaji A, Ardiles T, et al. Discovery and validation of cell cycle arrest biomarkers in human acute kidney injury. *Crit Care*. Feb 6 2013;17(1):R25. doi:10.1186/cc12503
5. Bihorac A, Chawla LS, Shaw AD, et al. Validation of cell-cycle arrest biomarkers for acute kidney injury using clinical adjudication. *Am J Respir Crit Care Med*. Apr 15 2014;189(8):932-9. doi:10.1164/rccm.201401-0077OC
6. Honore PM, Nguyen HB, Gong M, et al. Urinary Tissue Inhibitor of Metalloproteinase-2 and Insulin-Like Growth Factor-Binding Protein 7 for Risk Stratification of Acute Kidney Injury in Patients With Sepsis. *Crit Care Med*. Oct 2016;44(10):1851-60. doi:10.1097/CCM.0000000000001827

© 2022 Molinari L et al. *JAMA Network Open*.
