# Supplementary material for: Utility of Biomarkers for Sepsis-Associated Acute Kidney Injury Staging
Source: JAMA Netw Open. 2022 May 18;5(5):e2212709. doi: 10.1001/jamanetworkopen.2022.12709 (PMC9118077; doi:10.1001/jamanetworkopen.2022.12709)
Supplement: Supplement 2. — ProCESS and ProGReSS-AKI Investigators [file jamanetwopen-e2212709-s002.pdf]

Supplemental Online Content: Nonauthor Collaborators

*Indicates required information. Only first name, last name, and suffix will appear in PubMed.

***Group Name(s): ProCESS and ProGReSS-AKI Investigators**							
***First Name and Middle Initial(s)**	***Last Name**	***Suffix (eg, Jr, III)**	Academic Degrees	Institution	Location (city, state/province, country)	Role or Contribution, eg, chair, principal investigator	Group (if more than 1 Group listed in the byline) and/or Subgroup (eg, Steering Committee)
Christopher	Keener					Coordinating Center/Investigator	ProCESS/ProGReSS AKI
Nicole	Lucko					Investigator	ProGReSS AKI
Francis	Pike					Coordinating Center/Investigator	ProCESS/ProGReSS AKI
Sachin	Yende					Investigator	ProGReSS AKI
Amber E	Barnato					Coordinating Center	ProCESS
Tammy L	Eaton					Coordinating Center	ProCESS
Elizabeth	Gimbel					Coordinating Center	ProCESS
Kyle	Landis					Coordinating Center	ProCESS
Diana K	Stapleton					Coordinating Center	ProCESS
Lisa A	Weissfeld					Coordinating Center	ProCESS
Michael	Willochell					Coordinating Center	ProCESS
Kourtney A	Wofford					Coordinating Center	ProCESS
Erik	Kulstad			Advocate Christ Medical Center	Oak Lawn, IL	Site investigator	ProCESS
Hannah	Watts			Advocate Christ Medical Center	Oak Lawn, IL	Site investigator	ProCESS
Arvind	Venkat			Allegheny General Hospital	Pittsburgh, PA	Site investigator	ProCESS
Peter C	Hou			Brigham and Women’s Hospital	Boston, MA	Site investigator	ProCESS
Anthony	Massaro			Brigham and Women’s Hospital	Boston, MA	Site investigator	ProCESS
Siddharth	Parmar			Brigham and Women’s Hospital	Boston, MA	Site investigator	ProCESS
Alexander T	Limkakeng	Jr		Duke University Medical Center	Durham, NC	Site investigator	ProCESS
Kori	Brewer			East Carolina University	Greenville, NC	Site investigator	ProCESS
Theodore R	Delbridge			East Carolina University	Greenville, NC	Site investigator	ProCESS
Allison	Mainhart			East Carolina University	Greenville, NC	Site investigator	ProCESS
James R	Miner			Hennepin County Medical Center	Minneapolis, MN	Site investigator	ProCESS
Todd L	Allen			Intermountain Medical Center	Murray, UT	Site investigator	ProCESS
Colin K	Grissom			Intermountain Medical Center	Murray, UT	Site investigator	ProCESS
Stuart	Swadron			Los Angeles County + USC Medical Center	Los Angeles, CA	Site investigator	ProCESS
Steven A	Conrad			Louisiana State University Health Sciences Center	Shreveport, LA	Site investigator	ProCESS

Supplemental Online Content: Nonauthor Collaborators

*Indicates required information. Only first name, last name, and suffix will appear in PubMed.

***First Name and Middle Initial(s)**	***Last Name**	***Suffix (eg, Jr, III)**	Academic Degrees	Institution	Location (city, state/province, country)	Role or Contribution, eg, chair, principal investigator	Group (if more than 1 Group listed in the byline) and/or Subgroup (eg, Steering Committee)
Richard	Carlson			Maricopa Medical Center	Phoenix, AZ	Site investigator	ProCESS
Frank	LoVecchio			Maricopa Medical Center	Phoenix, AZ	Site investigator	ProCESS
Ednan K	Bajwa			Massachusetts General Hospital	Boston, MA	Site investigator	ProCESS
Michael R	Filbin			Massachusetts General Hospital	Boston, MA	Site investigator	ProCESS
Blair A	Parry			Massachusetts General Hospital	Boston, MA	Site investigator	ProCESS
Timothy J	Ellender			Methodist Research Institute	Indianapolis, IN	Site investigator	ProCESS
Andrew E	Sama			North Shore University Hospital	Manhasset, NY	Site investigator	ProCESS
Jonathan	Fine			Norwalk Hospital	Norwalk, CT	Site investigator	ProCESS
Soheil	Nafeei			Penn State Hershey College of Medicine	Hershey, PA	Site investigator	ProCESS
Thomas	Terndrup			Penn State Hershey College of Medicine	Hershey, PA	Site investigator	ProCESS
Margaret	Wojnar			Penn State Hershey College of Medicine	Hershey, PA	Site investigator	ProCESS
Ronald G	Pearl			Stanford University School of Medicine	Stanford, CA	Site investigator	ProCESS
Scott T	Wilber			Summa Health System	Akron, OH	Site investigator	ProCESS
Richard	Sinert			SUNY Downstate Medical Center	Brooklyn, NY	Site investigator	ProCESS
David J	Orban			Tampa General Hospital,	Tampa, FL	Site investigator	ProCESS
Jason W	Wilson			Tampa General Hospital,	Tampa, FL	Site investigator	ProCESS
Jacob W	Ufberg			Temple University Hospital	Philadelphia, PA	Site investigator	ProCESS
Timothy	Albertson			UC Davis Medical Center	Sacramento, CA	Site investigator	ProCESS
Edward A	Panacek			UC Davis Medical Center	Sacramento, CA	Site investigator	ProCESS
Sohan	Parekh			University Medical Center Brackenridge	Austin, TX	Site investigator	ProCESS
Scott R	Gunn			UPMC Presbyterian/Shadyside	Pittsburgh, PA	Site investigator	ProCESS
Jon S	Rittenberger			UPMC Presbyterian/Shadyside	Pittsburgh, PA	Site investigator	ProCESS
Richard J	Wadas			UPMC Presbyterian/Shadyside	Pittsburgh, PA	Site investigator	ProCESS
Andrew R	Edwards			University of Alabama at Birmingham	Birmingham, AL	Site investigator	ProCESS
Matthew	Kelly			University of Alabama at Birmingham	Birmingham, AL	Site investigator	ProCESS

Supplemental Online Content: Nonauthor Collaborators

*Indicates required information. Only first name, last name, and suffix will appear in PubMed.

***First Name and Middle Initial(s)**	***Last Name**	***Suffix (eg, Jr, III)**	Academic Degrees	Institution	Location (city, state/province, country)	Role or Contribution, eg, chair, principal investigator	Group (if more than 1 Group listed in the byline) and/or Subgroup (eg, Steering Committee)
Henry E	Wang			University of Alabama at Birmingham	Birmingham, AL	Site investigator	ProCESS
Talmage M	Holmes			University of Arkansas for Medical Sc	Little Rock, AR	Site investigator	ProCESS
Michael T	McCurdy			University of Maryland at Baltimore	Baltimore, MD	Site investigator	ProCESS
Craig	Weinert			University of Minnesota Medical Center	Fairview, MN	Site investigator	ProCESS
Estelle S	Harris			University of Utah Health Sciences Center	Salt Lake City, UT	Site investigator	ProCESS
Wesley H	Self			Vanderbilt University Medical Center	Nashville, TN	Site investigator	ProCESS
Diane	Dubinski			Vanderbilt University Medical Center	Nashville, TN	Site investigator	ProCESS
Carolyn A	Phillips			Washington Hospital Center	Washington, DC	Site investigator	ProCESS
Ronald M.	Migues			Washington Hospital Center	Washington, DC	Site investigator	ProCESS
